# Crosstalk between MSC-extracellular vesicles and *Olea europaea* leaf extract in encapsulated liposomal hydrogel: attenuation of neuroinflammation and brain neurotransmitter and memory impairment associated with obesity-induced high-fat diet

**DOI:** 10.3389/fphar.2025.1621092

**Published:** 2025-11-03

**Authors:** Doaa Ibrahim, Ioan Pet, Hoda S. Sherkawy, Haitham Eldoumani, Ola M. Fathy, Aya Elgamal, Heba S. A. Gharib, Asmaa A. Muhammed, Aya Sh. Metwally, Mirela Ahmadi, Daniela Puşcaşiu, Sherief M. Abdel-Raheem, Ahmed Abdelfattah-Hassan

**Affiliations:** ^1^ Department of Nutrition and Clinical Nutrition, Faculty of Veterinary Medicine, Zagazig University, Zagazig, Egypt; ^2^ Department of Biotechnology, Faculty of Bioengineering of Animals Resources, University of Life Sciences “King Mihai I” From Timisoara, Timisoara, Romania; ^3^ Department of Medical Biochemistry, Faculty of Medicine Aswan University, Aswan, Egypt; ^4^ Department of Anatomy and Embryology, Faculty of Veterinary Medicine, Mansoura University, Mansoura, Egypt; ^5^ Zagazig University Hospitals, Department of Biochemistry, Zagazig University, Zagazig, Egypt; ^6^ Department of Animal Histology and Anatomy, Faculty of Veterinary Medicine, Badr University in Cairo (BUC), Cairo, Egypt; ^7^ Behaviour and management of Animal, Poultry and Aquatics Department, Faculty of Veterinary Medicine, Zagazig University, Zagazig, Egypt; ^8^ Department of Medical Physiology, Faculty of Medicine, Aswan University, Aswan, Egypt; ^9^ Department of Pharmacology, Faculty of Veterinary Medicine, Aswan University, Aswan, Egypt; ^10^ Faculty of Medicine, “Victor Babes” University of Medicine and Pharmacy of Timisoara, Timisoara, Romania; ^11^ Department of Public Health, College of Veterinary Medicine, King Faisal University, Hofuf, Saudi Arabia; ^12^ Department of Veterinary Medicine, College of Applied and Health Sciences, A’Sharqiyah University, Ibra, Sultanate of Oman; ^13^ Department of Anatomy and Embryology, Faculty of Veterinary Medicine, Zagazig University, Zagazig, Egypt

**Keywords:** high-fat diet, brain dysfunction, memory deficit, neurotransmitter, membrane-bound extracellular vesicle, *Olea europaea* extract

## Abstract

Consumption of a high-fat diet (HFD) can trigger neuroinflammation, which may contribute to and increase the risk of neurodegenerative disease progression, ultimately leading to memory impairment. In the current study, the curative impact of a novel therapy combining *Olea europaea* leaf extract (OLE) encapsulated in a liposomal hydrogel (Lipo-OLE-Hydrogel) and mesenchymal stem cell-derived exosomes (MSC-Exo) was evaluated against HFD-induced brain dysfunction in a rat model. This assessment involved analyzing behavioral tasks, neurotransmitter levels, oxidative stress, neuroinflammation, endoplasmic reticulum-related markers, histopathological lesions, and immunostaining markers in brain tissues. The experimental groups were arranged for a 14-week study as follows: the first group received a control diet; the second group was fed an HFD; the third group was fed an HFD and treated with Lipo-OLE-Hydrogel; the fourth group was fed an HFD and treated with MSC-Exo; and the fifth group was fed an HFD and treated with both Lipo-OLE-Hydrogel and MSC-Exo. The findings of this study demonstrated that the neuroprotective effect of the combined Lipo-OLE-Hydrogel and MSC-Exo treatment was associated with a significant reduction in oxidative stress, as evidenced by the restoration of total antioxidant capacity and the marked decrease in oxidative biomarkers, including reactive oxygen species (ROS), H_2_O_2_, and malondialdehyde (MDA). The HFD-fed group exhibited greater glucose intolerance and increased body weight gain; however, these effects were significantly reversed in the group treated with the combination of Lipo-OLE-Hydrogel and MSC-Exo, even after long-term HFD induction. Impairments in behavioral tasks and memory were significantly improved in the group treated with the combined MSC-Exo and Lipo-OLE-Hydrogel therapy, with the MSC-Exo-alone group showing moderate improvement. The excessive inflammatory response and expression of endoplasmic reticulum stress-related genes were markedly attenuated following administration of Lipo-OLE-Hydrogel and MSC-Exo. This effect was mediated through the downregulation of pro-inflammatory and stress-related genes, including *IL-6, COX-2, iNOS, TLR2, TLR4, NLRP3, CHOP, JNK, XBP1,* and *ATF6*. The severity of the histopathological changes in the brain tissues, including the development of neoplastic epithelium and the invasion of some neoplastic masses, was significantly attenuated in the group treated with the combined Lipo-OLE-Hydrogel + MSC-Exo therapy. Immunohistochemical staining displayed that Bcl-2 protein expression was significantly restored to near normal levels, while TNF-α expression was significantly reduced in the group treated with the combined MSC-Exo and Lipo-OLE-Hydrogel therapy. Taken together, these findings highlight a novel and promising therapeutic approach that combines a natural protective agent (Lipo-OLE-Hydrogel) with regenerative medicine (MSC-Exo) to effectively combat the progression of HFD-induced neuroinflammation.

## 1 Introduction

People with an unhealthy high-fat diet (HFD) lifestyle are at a greater risk of developing obesity and chronic systemic metabolic diseases (Organization and Organization, 2018). The impaired ability of the body to utilize fat, coupled with an imbalance between caloric intake and energy expenditure in HFD-fed animals, suggests that a significant portion of the increased energy intake is stored as fat, leading to an expansion of body fat stores (Cavaliere et al., 2018). The brain is a highly stress-sensitive organ, particularly vulnerable to key pro-inflammatory components from dietary sources, including saturated fatty acids, cholesterol, added sugars, and refined grains (Christ et al., 2019). Long-term consumption of excessive amounts of fat can disrupt the homeostasis of the entire body, including brain health, memory, and mood-related behavior (de Paula et al., 2021).

Neuroinflammation in the brain is a key risk factor for neurodegenerative disorders, with its impact varying based on the severity of these conditions (Kumar 2018). The impact of a high-fat diet (HFD) on brain function is attributed to several independent mechanisms, including oxidative stress, activation of inflammatory signaling, immune cell recruitment, mitochondrial dysfunction, increased permeability of the blood–brain barrier, and impaired synaptic plasticity (Carraro et al., 2018; Liu et al., 2015). Furthermore, eating a high-fat diet results in an imbalance between fatty acid uptake and utilization, leading to the accumulation of toxic lipid species, resulting in increased reactive oxygen species (ROS) production and inflammation. A high-fat diet increases oxidative stress in the brain and impairs mitochondrial function, although the underlying mechanisms are not yet fully understood (Cavaliere et al., 2019). Additionally, oxidative stress contributes to brain damage and induces cellular injury, impairing learning and memory (Hazzaa et al., 2020). The mechanisms by which high-fat consumption affects neural function and the potential role of these mechanisms in promoting exaggerated non-homeostatic eating are not yet fully understood.

Olive products are a valuable source of polyphenols, especially oleuropein and its derivatives, such as the hydroxytyrosol oleuropein, which destroy free radicals and hinder the chemical oxidation of lipoproteins (Chiou et al., 2007). Olive leaves (*Olea europaea* leaves) provide a distinctive opportunity to investigate the effects of their polyphenols on metabolic profiles and cardiovascular and hepatic structure and function, due to their rich polyphenol content and low levels of oleic acid (Manna et al., 2004). The extract of olive leaves contains secoiridoids, such as oleuropein, dimethyloleuropein, ligostroside, and oleoside, as well as flavonoids like kaempferol, apigenin, and luteolin, along with phenolic compounds such as tyrosol, caffeic acid, and hydroxytyrosol. Among the many polyphenols in olive leaf extract, oleuropein has garnered significant attention in the food industry for its potential in formulating functional products with improved nutritional properties or increased shelf-life, thanks to its antimicrobial properties and ability to prevent food oxidation (Nunes et al., 2016). Additionally, oleuropein exhibits a wide range of pharmacological functions, including anti-inflammatory effects, reduced blood pressure, inhibition of platelet aggregation and eicosanoid production, scavenging of free radicals, and inhibition of 5- and 12-lipoxygenases (Visioli et al., 2002). Polyphenolic compounds in olive leaf extract (OLE) may be a promising option for managing excess weight, reducing the risk and progression of chronic diseases, and preventing various aspects of neurodegenerative conditions associated with HFD-induced obesity (Amel et al., 2016).

The neuroprotective effect of OLE is mainly due to the antioxidant properties of its polyphenolics. Due to these advantages, olive leaf extract (OLE) has been extensively studied for its numerous health benefits, including support for brain and heart health, modulation of lipid metabolism, preservation of antioxidant stores, as well as its anti-inflammatory and anti-tumor properties (Jimenez-Lopez et al., 2020; Tsartsou et al., 2019). However, the use of bioactive compounds from olive leaf extract (OLE) faces several challenges due to their low solubility, sensitivity to environmental conditions (e.g., temperature, pH, and light), and often poor sensory characteristics (González-Ortega et al., 2021). To overcome these challenges, nano-encapsulation techniques have been developed to enhance the usability and bioavailability of these compounds (Vinceković et al., 2017). Liposomes (Lipo) are vesicles composed of phospholipid bilayers, offering characteristics valuable to the food industry, such as the ability to encapsulate both amphiphilic and water- or lipid-soluble compounds, enable controlled release, and support large-scale production (Pimentel-Moral et al., 2018). Moreover, the application of hydrogel-based technologies in combination with liposomes can further enhance their mechanical stability and membrane integrity (Ibrahim et al., 2017). From a pharmacological perspective, the use of antioxidant therapies alone to target neuroinflammation associated with HFD-induced obesity has proven largely ineffective. However, combining liposomal olive leaf extract encapsulated in hydrogel (Lipo-OLE-Hydrogel) may offer a more effective strategy for managing excess weight, reducing the risk and progression of chronic diseases, and preventing various aspects of neurodegenerative conditions associated with HFD-induced obesity (Amel, Wafa, Samia, Yousra, Issam, Cheraif, Attia, and Mohamed, 2016). The neuroprotective effect of Lipo-OLE-Hydrogel is primarily attributed to the antioxidant properties of its polyphenols. However, from a pharmacological perspective, using antioxidant therapies alone to target neuroinflammation associated with HFD-induced obesity has proven ineffective so far.

Recently, mesenchymal stem cells (MSCs) have garnered significant interest as promising cell-based therapeutic approaches due to their ability to migrate and promote tissue repair (Guo et al., 2020). MSCs are key adult multipotent cells that possess self-renewal capacity and differentiation potential. They have been explored in cell replacement therapies for repairing damaged mesenchymal or ectodermal tissues, including neural tissue (Bagher et al., 2016). Mesenchymal stem cells or their exosomes can be considered a promising therapeutic strategy to increase antioxidant capacity and neurotrophin expression, while inhibiting pro-inflammatory cytokine secretion, key factors in neurodegenerative pathologies (Angeloni et al., 2020). The contribution of MSCs as nanotherapeutic agents to brain tissue repair and functional recovery has gained widespread interest, due to their ability to participate in the complex intercellular communication system (Merino-González et al., 2016).

Therefore, considering all of the above factors, the current study aimed to provide novel insights by investigating the injection of MSC-derived exosomes (MSC-Exo) combined with a nutritional therapeutic approach, specifically the ingestion of Lipo-OLE-Hydrogel. This innovative combined therapy (Lipo-OLE-Hydrogel + MSC-Exo) seeks to reverse the detrimental effects and health risks associated with the progression of HFD-induced neuroinflammation. Consequently, the therapeutic impact of Lipo-OLE-Hydrogel + MSC-Exo on HFD-induced brain dysfunction in rats was evaluated by assessing its modulatory effects on behavior-related tasks, neurotransmitters, neuroinflammation, oxidative stress, endoplasmic reticulum markers, and immunostaining-related markers in brain tissues.

## 2 Materials and methods

### 2.1 Encapsulation of *Olea europaea* leaf extract in liposomal hydrogel

The extract of *Olea europaea* leaves (OLE) was supplied by Sigma-Aldrich (product No. MFCD00131764). The analysis of the phenolic compounds in an extract of OLE (µg/kg) was conducted using high-performance liquid chromatography and is available in Supplementary Material 1. The liposome formulation was prepared using unsaturated 1,2-dioleoyl-sn-glycero-3-phosphocholine (DOPC, Mw 786.11, Sigma-Aldrich Product No. 4235-95-4) and cholesterol, following the lipid film hydration approach, combined with the freeze-thaw method, and subsequently processed using the extrusion method. In brief, optimal levels of lipid constituents (DOPC and cholesterol) were dissolved in CHCl_3_, while GLT1 was dissolved in MeOH. The mixture was then desiccated by rotary evaporation (BUCHI Labortechnik AG, Rotavapor R-200, Switzerland) and subjected to high vacuum (5 h) to eliminate any residual organic solvents, resulting in a thin lipid film.

Liposomes and OLE-loaded liposomes were prepared adopting the procedure that was described by Bonechi et al. (2012) and Song et al. (2022), using chloroform for lipids and ethanol for OLE (Mohan et al., 2014). Briefly, a proper amount of lipid components was dissolved in DOPC, cholesterol, and cationic galactosylated amphiphile (GLT1) in a flask and then dried by rotary evaporation (Rotavapor R-200, BUCHI Labortechnik AG, Flawil, Switzerland). A high vacuum was used overnight to remove organic solvent traces in order to attain a thin dry lipid film. The preparation of loaded liposomes and OLEs was dissolved in cationic galactosylated amphiphile and added to the lipid mixture, before film formation, to have a molar ratio of 1:8 OLEs/lipids and a final ratio of 1:1 (w/w) lipids/dry extract, respectively. Afterward, the lipid film was hydrated with a phosphate-buffered saline (PBS) (150 mM) solution to yield a liposomal suspension with a total lipid concentration of 10 mM. The aqueous suspension was vortex-mixed to fully detach the lipid film from the flask, and the resulting multilamellar vesicles (MLVs) were subjected to five cycles of freeze-thawing, alternating between liquid nitrogen and 50 °C. Size reduction of the MLVs was achieved by extrusion using a 10-mL liposome extruder (Genizer, Irvine, CA) under high pressure, passing the liposomal dispersion ten times through a polycarbonate membrane with a pore size of 100 nm (Whatman Nucleopore, Clifton, NJ) at a temperature above the T_m_, to produce small unilamellar vesicles (Bonechi et al., 2012).

**FIGURE 1:**
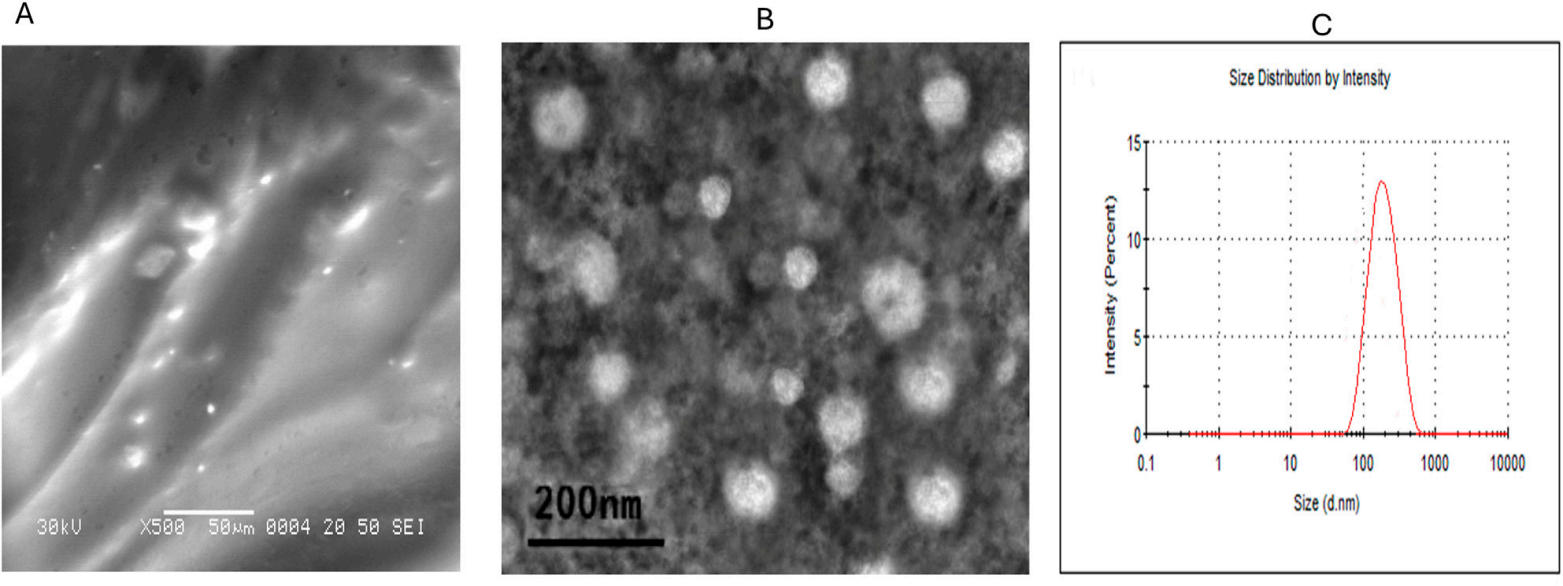
Characterization of olive leaves extract encapsulated liposomal hydrogel **(A)**, and mesenchymal stem cells derived exosomes **(B)** by transmission electron microscope and the particle size distribution **(C)** was achieved by dynamic light scattering.

The OLE-loaded liposome hydrogel was prepared by mixing the liposome OLE suspension with acrylamide, polyethylene glycol 147230 (Sigma-Aldrich, Saint Louis, MO, USA), dimethacrylate (437468, Sigma-Aldrich), ammonium persulfate, and tetramethylethylenediamine. The prepared liquid mixture was vortexed for 10 min and then kept in a vacuum chamber at room temperature for 4 h until complete gelation occurred. The morphological characterization of OLE-loaded liposome hydrogel (Lipo-OLE-Hydrogel) was performed using a field emission transmission electron microscope (JEM 2100F, JEOL, Japan, Figure 1A), operating at an accelerating voltage of 30 kV. The particle size distribution of Lipo-OLE-Hydrogel (Figure 1C) was achieved by dynamic light scattering (Zetasizer Nano ZS, Malvern, UK). The entrapment efficiency (EE%) of the prepared Lipo-OLE-Hydrogel was determined according to a previously established method (Shafique et al., 2017).

### 2.2 Experimental animals and study design

All animal experiment protocols were approved and conducted in compliance with the regulations of the Institutional Animal Care and Use Committee (ZU-IACUC/2021), Faculty of Veterinary Medicine, Zagazig University. Animals were acclimated for 1 week prior to the start of the experimental procedures and housed under controlled environmental conditions with a 12-h light/dark cycle, at a temperature of 23 °C ± 1 °C, humidity of 52% ± 5%, and *ad libitum* access to a standard chow diet (4.10 kcal/g) and water. All animal procedures, design, and reporting followed the ARRIVE guidelines. Fifty male Sprague–Dawley rats (178 ± 4 g) were divided into five groups (n = 10/group). Group 1, the control group, was fed the standard diet (10% from fat, D12450J, Research Diets, 10% Kcal Thermo Fisher Scientific) and had free access to water and feed. The obese rat groups (Groups 2, 3, 4, and 5) were fed a high-fat diet (HFD, 60% kcal, Research Diets Inc. D12492, Thermo Fisher Scientific). Group 2 received an HFD, Group 3 received an HFD plus *Olea europaea* leaf extract-loaded liposomal hydrogel (HFD + Lipo-OLE-Hydrogel) at a dose of 50 mg/kg/day via an oral route, Group 4 received an HFD and was injected with mesenchymal stem cell-derived exosomes (50 µg) (HFD + MSC-Exo), and Group 5 received an HFD and was treated with both Lipo-OLE-Hydrogel at a dose of 50 mg/kg/day via an oral route and MSC-Exo. Experimental rats were fed a high-fat diet (HFD) for 14 consecutive weeks and treated during the last 4 weeks.

Bone marrow (BM) mesenchymal stem cell (MSC)-derived exosomes (Exo) were isolated and characterized as previously reported (Alzahrani et al., 2018; El Nashar et al., 2021) from healthy non-diseased male rats, and exosomes obtained from rat BM-MSCs were isolated and characterized as previously performed (Abdelfattah-Hassan and Ibrahim, 2022; Alasmari et al., 2022) as described in Figure 1B. Briefly, when the cells reached 80% confluency after passage three, the MSC culture medium was detached, and the serum-free medium was replaced and allowed to incubate for 24 h. The collected conditioned medium was filtered (0.22 μm) to eliminate debris and dead cells, then centrifuged at 10,000 g at 4 °C for 30 min. In the next step, the supernatant was gathered. The supernatant was then exposed to ultracentrifugation at 100,000 g, 4 °C for 60 min to pellet the exosomes. After that, the prepared pellet was rinsed twice with ice-cold PBS, and ultracentrifugation was achieved for each wash to re-pellet the isolated exosomes. To quantify the amount of isolated exosomes, the protein content was measured by the standard Bradford assay, and then the isolated exosomes were adjusted to 100 µg protein in 200-µL aliquots and stored at −80 °C. Our protocol implemented a single-time freeze-thaw cycle. Intraperitoneal injection of 50 μg of exosomes in 200 μL of PBS was done once weekly. Throughout the experimental period, body weight and feed intake were monitored on a weekly basis.

### 2.3 Assessing the glucose intolerance test (GIT)

The GIT test was performed at 7 weeks and 14 weeks for all experimental groups. Rats from corresponding groups were fasted and anesthetized with pentobarbital (50 mg/kg, i.p). After that, all rats were injected with 25% glucose (2 g/kg body weight, i.p), then blood glucose levels were monitored at different time points: 10 min, 20 min, 40 min, 60 min, 80 min, 100 min, and 120 min post-glucose injection via a Glucometer (Accu-Chek, 2724288270372, Italy).

### 2.4 Behavioral tasks

#### 2.4.1 Assessing different behaviors and anxiety via open-field tests

Open-field tests were conducted using an open-field maze, consisting of a black wooden box (50 cm × 50 cm × 30 cm) with black wooden walls. Prior to the test, the rats were brought to the testing room for 5–20 min to acclimatize. Each rat was gently handled and placed in the corner of the box, facing the corner. The subject rat was allowed free and uninterrupted movement in the maze for 3 min. All animals were video-recorded to automatically collect behavioral data, involving the number of boxes entered, the number of rears, the number of fecal boli, and the occurrence of stretch-attend postures (Deacon and Rawlins, 2006).

#### 2.4.2 Assessing working memory and learning via the modified Barnes maze test and the modified T-maze

The modified Barnes maze test was conducted using a white, circular wooden platform elevated 90 cm from the ground. The platform had 18 equally spaced holes (diameter = 10 cm) along the perimeter, with only one hole leading to the escape tunnel. In the center of the maze, a black wooden start chamber was placed, and a removable black escape box was positioned beneath one of the platform’s holes. Habituation was conducted 24 h prior to the trial for all rats, with trials lasting 5 days (2 trials/day for 5 days, each lasting 3 min or until the rat entered the escape box). Latency to reach the escape and error times were calculated accordingly (Gawel et al., 2019).

In the modified T-maze test, a manually operated T-maze with a central partition and guillotine doors at each goal arm was constructed to assess spontaneous alternation. Briefly, no habituation to the maze was allowed as the novelty of the maze itself motivated spontaneous exploration and alternation. For each rat, one sample trial and five choice runs were performed per day for 2 days, amounting to a total of 12 trials per rat and a total of ten possible alterations. In each trial, the rat was placed in the starting area at the base of the T-maze and allowed to choose one of the goal arms. The percentage of correct choices (alternations) per animal was calculated as follows (Deacon and Rawlins, 2006)

$$=\frac{\text{Number}\;\text{of}\;\text{alternations}\left(\text{Correct}\;\text{choices}\right)}{\text{Total}\;\text{possible}\;\text{alternations}\;}\;x\;100$$

#### 2.4.3 Novelty-suppressed feeding test and home cage feeding

The novelty-suppressed feeding test (NSFT) was conducted as follows (Warner-Schmidt and Duman, 2007). A small amount of feed pellets was offered to the rats (food-deprived overnight) from each respective group, and then they were placed in an open field with both the control and HFD groups positioned in the center to avoid any olfactory bias. At the time of the test, animals were subjected to the open field for the first time (novelty) and permitted to explore for 15 min. The time taken for the animal to approach and take its first bite of the feed was recorded as latency to feed. Also, the home cage food intake (HCF) of the different groups was recorded over a 10-min period.

### 2.5 Sampling collection

At the end of the experiment, rats were anesthetized with intravenous 30 mg/kg bodyweight ketamine hydrochloride and then euthanized by cervical dislocation. Blood was collected in a plain tube without an anticoagulant, then centrifuged at 4 °C at 4,000 rpm for 10 min for serum collection. For preparation of brain tissue homogenates, brain tissues were collected, rinsed with cold saline, and macerated in phosphate-buffered saline (PBS, ten volumes/weight). Then, the homogenate was centrifuged at 1,500 × g for 10 min, and the clear supernatant was gathered for the measurements of neurotransmitters and oxidative stress biomarkers. For gene expression (RT-qPCR), the brain tissues were removed and directly stored at −80 °C. The remaining brain tissues were collected for histopathological and immunohistochemical examination and fixed in 10% formalin buffer.

### 2.6 Serum biochemical-related parameter assessment

The serum concentrations of ALT, AST, urea, and creatinine were assessed via commercial kits (Sigma-Aldrich, MAK080, MAK006, MAK052, MAK055, respectively). Quantitative estimations of triglycerides, cholesterol, high-density lipoprotein (HDL), and low-density lipoprotein (LDL) were done by an ELISA kit from MyBioSource, Catalog Number. MBS726298, BS722885, MBS266554, and MBS702165, respectively. Quantitative analysis for leptin was done using an ELISA kit from Sigma-Aldrich (RAB0334-1KT). Serum cortisol was measured as stated in the method established by Chen X-M. et al. (2016) via sandwich MyBioSource kits.

### 2.7 Neurotransmitters and oxidant/antioxidant status of brain tissues

The concentration of serotonin was estimated via the Quantitative ELISA kit from MyBioSource, Cat. No. MBS166089. The concentrations of dopamine and glutamate were determined using a Competitive ELISA kit from MyBioSource, MBS725908 and MBS756400, respectively. The brain tissue homogenates were used for the malondialdehyde (MDA) and total antioxidant capacity (TAC) estimation (CAT.NO MBS508035) and (CAT.NO MBS169313), respectively, following the manufacturer’s instructions. ELISA kits from MyBioSource (San Diego, USA) were used for estimation of superoxide dismutase (SOD, CAT. NO. MBS036924), catalase (CAT, CAT. NO. MBS006963), and glutathione peroxidase (GPX, CAT. NO. MBS028183) in brain tissues. The brain ROS content was determined via an enzyme-linked immunosorbent assay (ELISA) kit specific to ROS (MBS039665, MyBioSource; San Diego, USA) following the manufacturer’s instructions. Brain hydrogen peroxide (H_2_O_2_) levels were estimated according to the procedures previously detailed by Loreto and Velikova (2001), and their values were expressed as μmoL/g of tissue.

### 2.8 RNA extraction and quantitative real-time PCR

Total RNA was isolated from the collected brain tissues utilizing RNeasy Mini kits (Qiagen, CA, USA) in accordance with the protocol guidelines. The purity and quantity of the extracted RNA were assessed via a NanoDrop™ ND-1000 Spectrophotometer (Thermo Fisher Scientific Inc., Waltham, MA, USA). Extracted RNA was reverse-transcribed into cDNA using a Maxima First-Strand cDNA Synthesis kit (Thermo Scientific, USA). Synthesis of cDNA was done at a 60-min incubation at 37 °C for activation, then heating to 95 °C for 5 min, and ultimately, a holding temperature of 4 °C (thermal cycler with Averitt 96-well, Applied Biosystems, Foster City, CA, United States). Brain tissues were used for evaluating the expression of genes encoding cytokines [interleukin (*IL*)-*1β, IL-6,* tumor necrosis factor α (*TNF-α*)] and other inflammatory markers [cyclooxygenase-2 (*COX-2*), toll-like receptors (*TLR2 and TLR4)* and inducible nitric oxide synthase (*iNOS*)] and pyrin domain-containing protein 3 (NLRP3) inflammasome, anti-apoptotic genes and pro-apoptotic genes [C/EBP homologous protein (*CHOP*), c-Jun N-terminal kinase (*JNK*), eukaryotic initiation factor 2α (*eIF2α*), activating transcription factor (*ATF-6*), X-box-binding protein-1 (*XBP1*)], immunoglobulin-binding protein (*BIP*), and oxidative stress-related genes [glutathione peroxidase (*GSH-Px*), catalase, superoxide dismutase (*SOD*), heme oxygenase-1 (*HO-1*), and nuclear factor erythroid 2-related factor 2 (*Nrf2*]. Stress-induced genes [Cytochrome P450 Family 11 Subfamily B Member 1 (*CYP11B1*), cytochrome c, and caspase-3]. The expression levels of inflammation and apoptosis-associated target genes were assessed through quantitative real-time PCR (qRT-PCR) utilizing a SYBR green kit (Qiagen, Hilden, Germany) and precise primers for the respective marked genes. All PCRs were accomplished in triplicate using the Stratagene MX3005P real-time PCR system (Stratagene, La Jolla, CA, United States). The gene expression profiles were considered employing the ^2–ΔΔ^CT method (Livak and Schmittgen, 2001), where β-actin served as a stable endogenous control gene. The sequences of the gene-target primers are listed in Table 1.

**TABLE 1:** Primer sequences utilized for rRT-PCR analysis of targeted gene expression.

Target gene	Primer sequence (5′–3′)	Accession No.
*IL-1β*	F-TGACAGACCCCAAAAGATTAAGG R-CTCATCTGGACAGCCCAAGTC	NM_031512.2
*IL-6*	F-CCACCAGGAACGAAAGTCAAC R-TTGCGGAGAGAAACTTCATAGCT	NM_012589.2
*TNF-α*	F-CAGCCGATTTGCCATTTCA R-AGGGCTCTTGATGGCAGAGA	L19123.1
*TLR2*	F-CGCTTCCTGAACTTGTCC R-GGTTGTCACCTGCTTCCA	XM_008761102.3
*TLR-4*	F- TCCCACTCGAGGTAGGTGTT R-TTGTTAAGCTTATAAATCATGCGGCCTCAGG	NM_019178.2
*Nrf-2*	F - GGTTGCCCACATTCCCAAAC R - GGCTGGGAATATCCAGGGCA	NM_031789.2
*HO-1*	F - CCCAGAGGCTGTGAACTCTG R - AGGCCCAAGAAAAGAGAGCC	NM_012580.2
*NLRP3 inflammasome*	F-CCAGGGCTCTGTTCATTG R-CCTTGGCTTTCACTTCG	XM_039085397.1
CYP11B1	ATGCCATCCATGCCAACTCA AGGACTAGAGCTGGGCATCA	NM_012537.3
*iNOS*	F-ACC TTC CGG GCA GCC TGT GA R-CAA GGA GGG TGG TGC GGC TG-3′	NM_012611
*COX-2*	F-GCT CAG CC ATA CAG CAA ATC C R-GGG AGT CGG GCA AT CAT CAG	NM_017232
*Caspase-3*	F-GCA GCT AAC CTC AGA GAG ACA TTC R-ACG AGT AAG GTC ATT TTT ATT CCT GACTT	NM_012922
*Bcl-2*	F-TGC GCT CAG CCC TGTG R-GGT AGC GAC GAG AGA AGT CATC	NM_016993
*BAX*	F-CAA GAA GCT GAG CGA GTG TCT R-CAA TCA TCC TCT GCA GCT CCA TAT T	NM_017059
*Cytochrome C*	F-TTT GAA TTC CTC ATT AGT AGC TTT TTTGG R-CCA TCC CTA CGC ATC CTT TAC	NM_012839
*SOD*	F: AGCTGCACCACAGCAAGCAC R: TCCACCACCCTTAGGGCTCA	NM_017051.2
*CAT*	F: ACGAGATGGCACACTTTGACAG R: TGGGTTTCTCTTCTGGCTATGG	NM_012520.2
*GSH-Px*	F: AAGGTGCTGCTCATTGAGAATG R: CGTCTGGACCTACCAGGAACT	NM_030826.4
*CHOP*	F 5′- CACAAGCACCTCCCAAAG -3′ R 5′- CCTGCTCCTTCTCCTTCAT -3′	NM_001109986.1
*XBP-1*	F 5′- TTACGAGAGAAAACTCATGGGC -3′ R 5′-GGGTCCAACTTGTCCAGAATGC-3′	NM_001004210.2
*EIF-2*	F 5′- CTTTCCGGGACAAGATGGCG -3′ R 5′- CTCTGTGAAGTGTGGGGGTC -3′	NM_001399818.1
*ATF-6*	F 5′- AAGTGAAGAACCATTACTTTATATC -3′ R 5′-TTTCTGCTGGCTATTTGT-3′	NM_001107196.1
*BIP*	F 5′- AACCAAGGATGCTGGCACTA -3′ R 5′-ATGACCCGCTGATCAAAGTC -3′	NM_013083.2
*JNK*	F 5′- AGTGTAGAGTGGATGCATGA -3′ R 5′- ATGTGCTTCCTGTGGTTTAC -3′	NM_053829.2
β-actin	F-CGCAGTTGGTTGGAGCAAA R-ACAATCAAAGTCCTCAGCCACAT	V01217.1
GAPDH	F-TGC TGG TGC TGA GTA TGT CG-3′ R-TTG AGA GCA ATG CCA GCC -3′	NM_017008

Activating transcription factor (*ATF-6*), Associated x protein (*Bax)*, B cell lymphoma-2 (*Bcl-2)*, Bcl-2, C/EBP, Catalase (CAT), c-Jun N-terminal kinase (*JNK*), Cyclooxygenase-2 *(COX-2)*, Cyclo-oxygenase-2 *(COX-2)*, Eukaryotic initiation factor 2α (eIF2α), Glutathione peroxidase (GSH-Px), Glyceraldehyde 3-phosphate dehydrogenase (GAPDH), Heme oxygenase-1 (HO-1), Homologous protein (*CHOP*), IL-6, Immunoglobulin-binding protein (*BIP*), Inducible nitric oxide synthase *(iNOS)*, Interleukin (IL)-1β, Nuclear factor erythroid 2-related factor 2 (Nrf2), Pyrin domain-containing protein 3 (NLRP3) inflammasome, Superoxide dismutase (SOD), Toll-like receptors (TLR), Tumor necrosis factor α (*TNFα)*, X-box-binding protein-1 (*XBP1*).

### 2.9 Histopathological analysis

Brain tissue samples were collected from the cerebral cortex region and preserved in 10% neutral buffered formalin for 24 h, followed by dehydration in graded ethanol, embedding in paraffin, and sectioning with a microtome instrument (Leica RM 2155, England). Subsequently, the prepared tissue sections (5-μm) were then prepared for staining employing conventional techniques of a hematoxylin and eosin (H&E) protocol and further examined under light microscopy (Ibrahim et al., 2021). The histopathological samples were graded for brain lesions, such as distorted crypt architecture, epithelial damage, and inflammatory cell infiltration (Wirtz et al., 2007).

### 2.10 Immunohistochemical detection

Paraffin sections from brain tissues were stained by immunohistochemistry (IHC) according to Hsu et al. (1981) using Anti-TNF alpha antibody [TNFA/1172] (ab220210) at 4 μg/mL and Anti-Bcl2 Rabbit polyclonal antibody (ab194583) at a dilution of 1:100 (Cambridge, UK, Abcam). The brain sections from all experimental groups were dewaxed and hydrated. Staining was then performed according to the manufacturer’s protocols and by using the DAB chromogenic agent (Expose mouse- and rabbit-specific HRP/DAB detection kit, Abcam; Ready-to-use; Cat. #: ab80436). Counterstaining by hematoxylin was done. For each antigen, three immuno-labeled sections were analyzed per animal (N = 5 animals per group). All images of the tissue sections subjected to immunohistochemistry (IHC) staining were acquired using a Swift microscope correlated with a Swift ordinal camera. For quantitative analysis, we selected five descriptive areas, including both positive cell areas and areas without expression. If a tissue section had areas with both low abundance and high abundance of stained cells, both areas were chosen as descriptive areas and integrated into the analysis. Individual cells were recognized by strong brown stains and manually calculated. The cell counts were quantified three times for each area, and all captured images were examined blindly.

## 3 Results

### 3.1 Biochemical assessment

Table 2 described the changes in lipid profile, leptin hormone level, liver, and kidney function markers in high-fat diet (HFD)-induced obese rats in response to the Lipo-OLE-Hydrogel + MSC-Exo treatment. Remarkably, all groups fed an HFD and treated either with MSC-Exo alone or in combination with Lipo-OLE-Hydrogel showed significantly decreased (*P* < 0.05) levels of triglycerides, total cholesterol, LDLP-C, leptin hormone, and cortisol. HDL-C levels were notably higher in the groups treated with MSC-Exo or the combined Lipo-OLE-Hydrogel + MSC-Exo therapy, which remained within the normal range, followed by the group treated with Lipo-OLE-Hydrogel alone, compared to the untreated HFD group. The most pronounced reductions in ALT, AST, urea, and creatinine were observed in the group receiving the combined Lipo-OLE-Hydrogel + MSC-Exo treatment.

**TABLE 2:** Changes in lipid profiles, leptin hormones, and liver and kidney functions in serum post treatment with Lipo-OLE-Hydrogel and/or MSC-Exo in high-fat diet (HFD)-induced obese rats.

	Experimental groups						
Parameter	Control	HFD	HFD +Lipo-OLE-Hydrogel	HFD + MSC-Exo	HFD + Lipo-OLE-Hydrogel + MSC-Exo	*P-*value	SEM
Triglycerides (mg/dL)	198.87^e^	404.43^a^	265.42^b^	245.11^c^	236.11^d^	<0.001	0.29
Total cholesterol (mg/dL)	49.76^e^	182.64^a^	111.57^b^	96.00^c^	86.98^c^	<0.001	0.18
HDLP (mg/dL)	45.79^d^	120.55^a^	88.78^b^	66.78^c^	63.87^c^	0.03	0.23
LDLP (mg/dL)	56.76^d^	175.55^a^	136.78^b^	110.87^c^	108.98^c^	0.04	0.32
Leptin (ng/mL)	0.88^d^	5.65^a^	2.23^b^	2.15^bc^	2.02^c^		
ALT (U/L)	7.67^e^	18.98^a^	13.54^b^	10.97^c^	8.87^d^	0.04	0.17
AST (U/L)	22.36^d^	98.54^a^	59.98^b^	56.76^b^	37.87^c^	0.02	0.23
Urea (U/L)	1.50^d^	2.98^a^	1.88^b^	1.79^bc^	1.62^c^	0.01	0.12
Creatinine (U/L)	0.45^d^	1.85^a^	1.23^b^	1.26^b^	0.87^c^	<0.001	0.3
Cortisol (nmol/L)	6.43	12.54	9.87	7.78	7.15	<0.001	0.09

^a-e^ Mean values with different letters in the same column differ significantly at *P* < 0.05. HDLP: high-density lipoprotein, LDLP: low-density lipoprotein, ALT: alanine transaminase, AST: aspartate transaminase. Control (group fed the standard diet); HFD (group fed a high-fat diet (60% Kcal); HFD + Lipo-OLE-Hydrogel [group fed a high-fat diet (60% Kcal) and treated with olive-leaf-encapsulated liposomal hydrogel]; HFD + MSC-EXO (group fed a high-fat diet and treated with mesenchymal stem cell exosomes); HFD + Lipo-OLE-Hydrogel + MSC-EXO (group fed a high-fat diet and treated with olive-leaf-encapsulated liposomal hydrogel); HFD and mesenchymal stem cell exosomes.

### 3.2 Assessment of neurotransmitters, oxidative stress, and antioxidant biomarkers in brain tissues

Notably, all HFD-fed groups showed significantly increased levels of MDA, H_2_O_2_, and ROS, along with reduced total antioxidant capacity (TAC) and antioxidant enzyme activity, compared to the control group (Table 3). Remarkably, the group treated with Lipo-OLE-Hydrogel + MSC-Exo exhibited significantly lower levels of MDA, H_2_O_2_, and ROS (31.00, 1.90, and 41.26, respectively) compared to the untreated HFD group (56.98, 5.11, and 132.63, respectively), alongside a notable increase in TAC (69.08 vs. 29.70). Groups treated with MSC-Exo alone or in combination with Lipo-OLE-Hydrogel also displayed significantly diminished (*P* < 0.05) serotonin levels, unlike the untreated HFD group. While HFD elevated dopamine and glutamate levels, treatment with Lipo-OLE-Hydrogel + MSC-Exo significantly (*P* < 0.05) reduced these levels, irrespective of continued HFD feeding.

**TABLE 3:** Changes in neurotransmitters, oxidative stress, and antioxidant biomarkers in brain tissues post treatment with Lipo-OLE-Hydrogel and/or MSC-Exo in high-fat diet (HFD)-induced obese rats.

	Experimental groups						
Parameter	Control	HFD	HFD +Lipo-OLE-Hydrogel	HFD + MSC-Exo	HFD + Lipo-OLE-Hydrogel + MSC-Exo	*P-*value	SEM
Serotonin (ng/mL)	97.56^d^	32.87^a^	46.76^b^	83.98^c^	87.87^c^	0.04	0.17
Dopamine (ng/mL)	43.30^d^	111.51^a^	88.98^b^	73.91^b^	77.87^c^	0.02	0.23
Glutamate (ng/mL)	31.84^d^	78.12^a^	53.11^b^	48.31^bc^	44.56^c^	0.01	0.12
MDA (μmoL/g tissue)	23.11^d^	56.98^a^	34.43^b^	31.54^c^	31.00^c^	<0.001	0.3
H_2_O_2_ (μmoL/g tissue)	1.23^d^	5.11^a^	2.11^b^	1.98^bc^	1.90^c^	<0.001	0.29
ROS (μmoL/g tissue)	29.70^d^	132.63^a^	59.12^b^	47.00^bc^	41.26^c^	<0.001	0.18
TAC (mol Trolox equivalent/g tissue)	29.65^c^	16.22^d^	63.60^b^	67.70^b^	69.08^a^	<0.001	0.23
GSH-PX (U/mg)	19.16^c^	9.54^d^	31.98^b^	32.39^b^	34.34^a^	<0.001	0.11
SOD (U/mg)	34.65^d^	6.78^e^	40.65^c^	43.65^b^	44.98^a^	0.02	0.14
CAT (U/mg)	39.87^d^	13.76^e^	53.56^c^	54.66^b^	56.87^a^	0.03	0.23

^a-e^ Mean values with different letters in the same column differ significantly at *P* < 0.05.

CAT: catalase, GSH-PX: glutathione peroxidase, H_2_O_2_: hydrogen peroxide, MDA: malondialdehyde, ROS: reactive oxygen species, SOD: superoxide dismutase, TAC: total antioxidant capacity. Control (group fed the standard diet); HFD (group fed a high-fat diet (60% Kcal); HFD + Lipo-OLE-Hydrogel [group fed a high-fat diet (60% Kcal) and treated with olive-leaf-encapsulated liposomal hydrogel]; HFD + MSC-Exo (group fed a high-fat diet and treated with mesenchymal stem cell exosomes); HFD + Lipo-OLE-Hydrogel +MSC-Exo (group fed a high-fat diet and treated with olive-leaf-encapsulated liposomal hydrogel and mesenchymal stem cell exosomes).

### 3.3 Assessment of the changes in body weight and blood glucose intolerance test of HFD-fed rats in response to MSC-Exo+ Lipo-OLE-Hydrogel treatment

**FIGURE 2:**
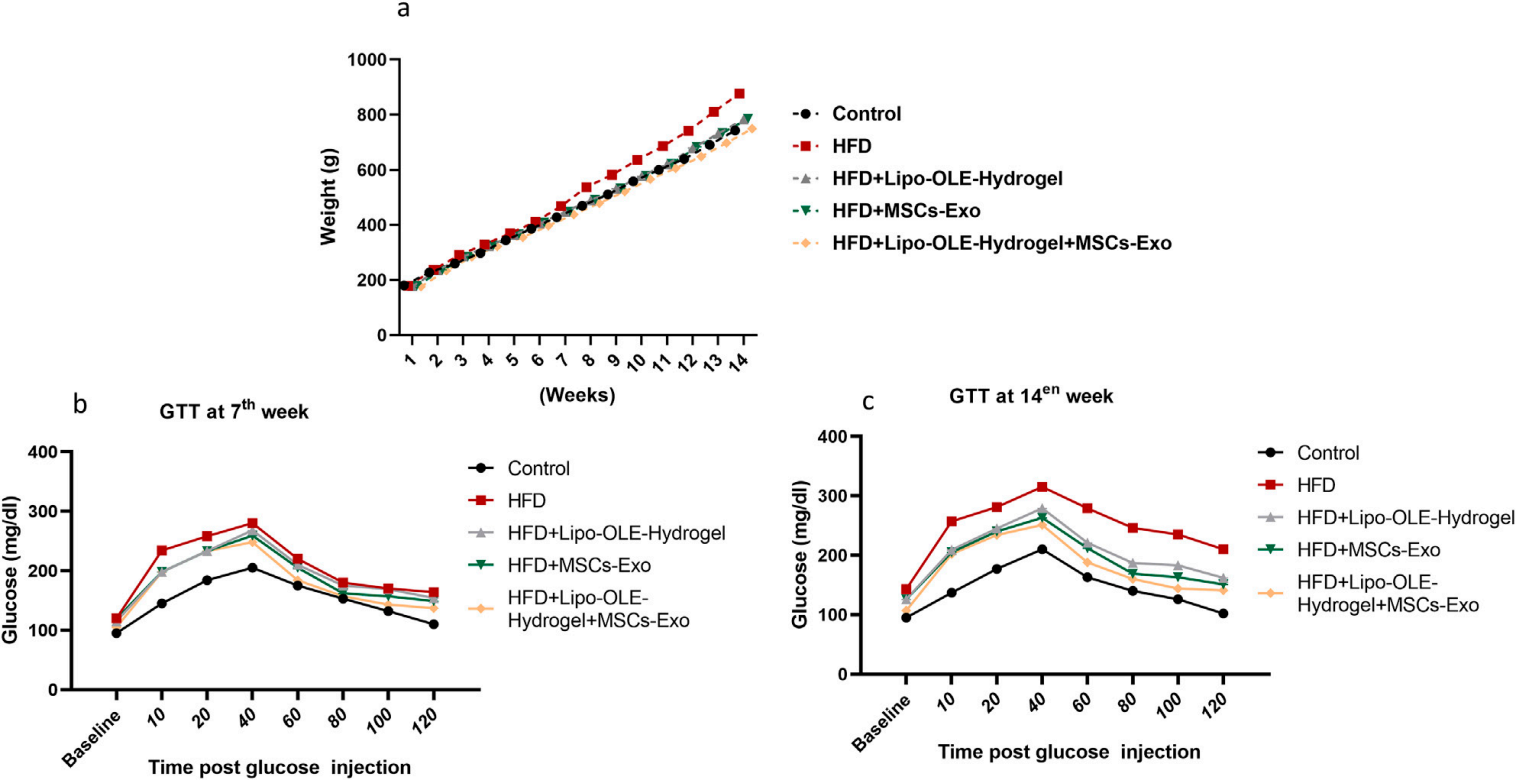
Changes of body weight **(a)** and blood glucose intolerance test at the 7th week **(b)** and the 14th week **(c)** in response to Lipo-OLE-Hydrogel and/or MSC-Exo treatment. Control (group fed the standard diet); HFD [group fed a high-fat diet (60% Kcal)]; HFD +Lipo-OLE-Hydrogel (group fed a high-fat diet (60% Kcal and treated with olive-leaf-encapsulated liposomal hydrogel); HFD + MSC-Exo (group fed a high-fat diet and treated with mesenchymal stem cell exosomes); HFD + Lipo-OLE-Hydrogel + MSC-Exo (group fed a high-fat diet and treated with olive-leaf-encapsulated liposomal hydrogel and mesenchymal stem cell exosomes). Data are expressed as means ± SE. Bars with altered letters imply significant variations (*P* < 0.05).

The effects of Lipo-OLE-Hydrogel + MSC-Exo combined therapy on ameliorating the increase in the rats’ body weight and restoring the blood glucose intolerance test are illustrated in Figure 2. Rats fed an HFD exhibited a significant increase in body weight (*P* < 0.05) after the 14-week experimental period compared to control rats on the standard diet. However, this weight gain was significantly attenuated in the group treated with Lipo-OLE-Hydrogel + MSC-Exo, with final body weight approaching that of the control group (749 g vs. 743 g, respectively; Figure 2a). Following glucose administration, blood glucose levels initially increased in both control and HFD-fed rats. A marked reduction in glucose levels was observed by 40 min post-injection in the control and Lipo-OLE-Hydrogel + MSC-Exo-treated groups, with glucose levels nearly returning to baseline by 120 min in the control group (Figure 2b, c). HFD-fed rats exhibited impaired glucose clearance, with significant differences from the control group evident as early as 7 weeks and worsening by 14 weeks. Fasting baseline glucose levels were consistently higher in HFD-fed rats than in the controls at both time points. Remarkably, after 14 weeks, the group treated with Lipo-OLE-Hydrogel + MSC-Exo demonstrated the lowest post-injection glucose levels at 120 min (120 mg/dL vs. 210 mg/dL in untreated HFD rats; *P* < 0.05), indicating improved glucose tolerance.

### 3.4 Assessment of behavioral tasks of HFD-fed rats in response to Lipo-OLE hydrogel + MSC-Exo treatment in the open field test

**FIGURE 3:**
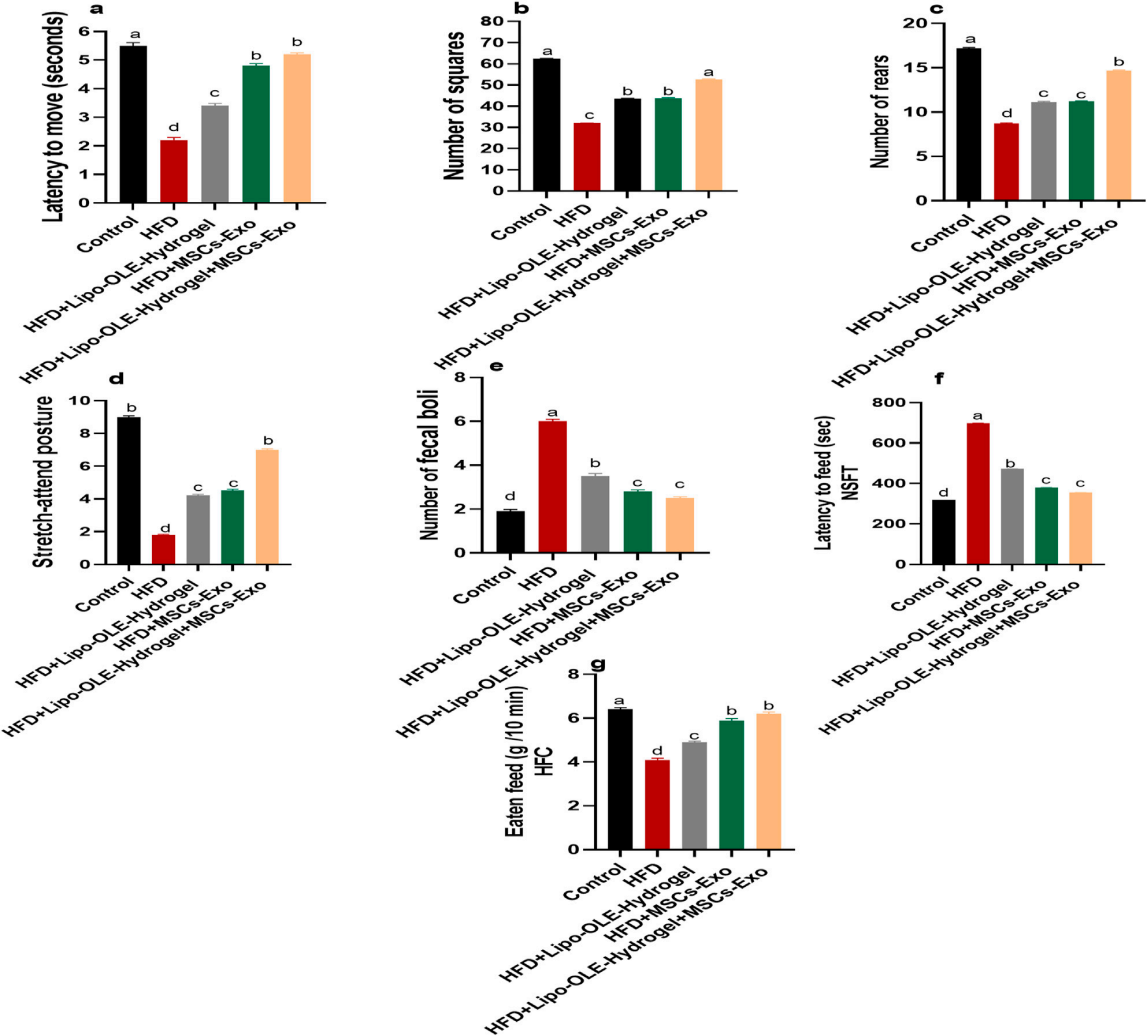
Changes in behavioral and anxiety tasks: latency to move **(a)**, the number of boxes entered by the rat **(b)**, the number of rears **(c)**, stretch-attend posture **(d)**, and the number of fecal boli **(e)**, latency to feed **(f)**, and feed eaten in home cage **(g)** in response to Lipo-OLE-Hydrogel and/or MSC-Exo treatment. Control (group fed the standard diet); HFD [group fed a high-fat diet (60% Kcal)]; HFD +Lipo-OLE-Hydrogel [group fed a high-fat diet (60% Kcal) and treated with olive-leaf-encapsulated liposomal hydrogel]; HFD + MSC-Exo (group fed a high-fat diet and treated with mesenchymal stem cell exosomes); HFD + Lipo-OLE-Hydrogel + MSC-Exo (group fed a high-fat diet and treated with olive-leaf-encapsulated liposomal hydrogel and mesenchymal stem cell exosomes). Data are expressed as means ± SE. Bars with altered letters imply significant variations (*P* < 0.05).

HFD-fed, non-treated rats showed a significant increase (*P* < 0.05) in the time taken to move from the outer zone and enter the inner boxes, along with a significant decrease (P < 0.05) in the number of squares entered, rearing behavior, and stretch-attend postures compared to the control group fed the standard diet (Figures 3b–d). However, these behavioral changes were greatly restored in the group treated with Lipo-OLE-Hydrogel + MSC-Exo when compared with the HFD non-treated group. Notably, the increased fecal boli production observed in the untreated HFD group was significantly reduced (*P* < 0.05) in HFD-fed rats treated with MSC-Exo or Lipo-OLE-Hydrogel + MSC-Exo (Figure 3e). HFD also significantly increased latency to feed in the novelty-suppressed feeding test (NSFT) compared to controls (control: 319.7 s; HFD: 698.6 s; Figure 3f). This latency was significantly reduced (*P* < 0.05) in HFD-fed rats treated with MSC-Exo or Lipo-OLE-Hydrogel + MSC-Exo (379.9 s and 354.9 s, respectively), indicating reduced anxiety-like behavior. Additionally, food intake during the 10-min home cage feeding test was significantly lower in the untreated HFD group (4.1 g) than in the control group (6.4 g). However, treatment with Lipo-OLE-Hydrogel, MSC-Exo, or the combined therapy significantly increased food intake (5.9 g and 6.2 g, respectively; *P* < 0.05).

### 3.5 Assessment of working memory and learning of HFD-fed rats in response to Lipo-OLE-Hydrogel +MSC-Exo treatment

**FIGURE 4:**
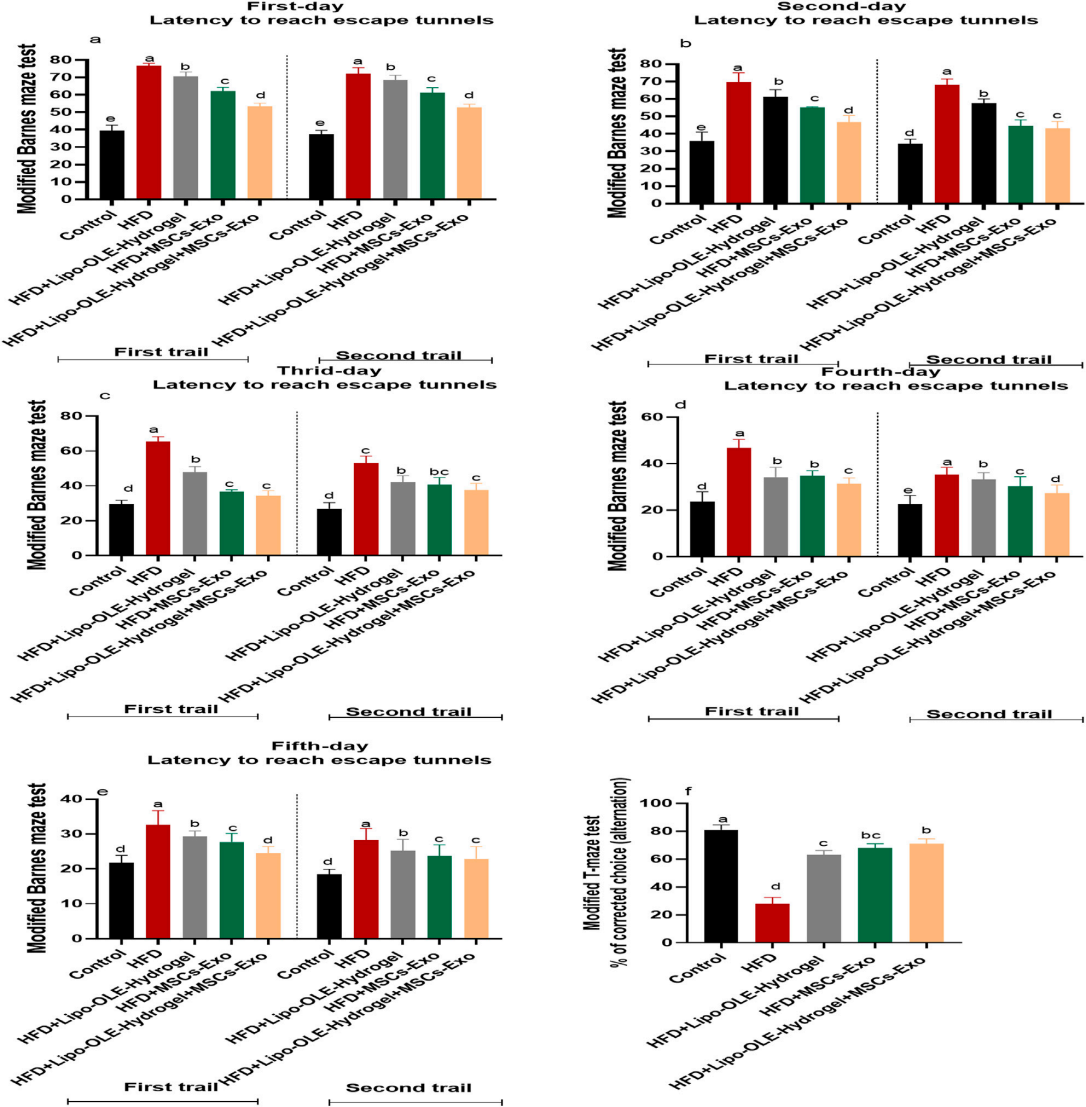
Changes in working and learning memory of HFD-fed rats in response to Lipo-OLE-Hydrogel + MSC-Exo treatment. Modified Barnes maze test **(a–e)** and modified T-maze test **(f)**. Control (group fed the standard diet); HFD [group fed a high-fat diet (60% Kcal]; HFD +Lipo-OLE-Hydrogel [group fed a high-fat diet (60% Kcal) and treated with olive-leaf-encapsulated liposomal hydrogel]; HFD + MSC-Exo (group fed a high-fat diet and treated with mesenchymal stem cell exosomes); HFD + LIPO-OLE-Hydrogel + MSC-Exo (group fed a high-fat diet and treated with olive-leaf-encapsulated liposomal hydrogel and mesenchymal stem cell exosomes). Data are expressed as means ± SE. Bars with altered letters imply significant variations (*P* < 0.05).

Modified Barnes maze test: Rats that received an HFD and were not treated spent more time to reach escape tunnel than (*p* < 0.05) than the control group. Meanwhile, rats in the MSC-Exo or Lipo-OLE- Hydrogel + MSC-Exo treated groups took less time to reach the escape tunnel (*p* < 0.05), but their times were still significantly higher than those of the control group fed the standard diet in all tested trials for 3 days (Figure 4a).

Modified T-maze test: The T-maze scores for rats that received an HFD and were not treated was significantly lower than those of the control group (*p* < 0.05), while all groups treated with MSC-Exo or Lipo-OLE- Hydrogel + MSC-Exo displayed a significantly increased score (63, 68%, and 71%, respectively) compared to the non-treated (28%) group, but their scores were still significantly lower than those of the control group fed the standard diet (81%) (Figures 4b–d).

### 3.6 Assessment of antioxidant markers of HFD-fed rats in response to Lipo-OLE Hydrogel + MSC-Exo treatment

**FIGURE 5:**
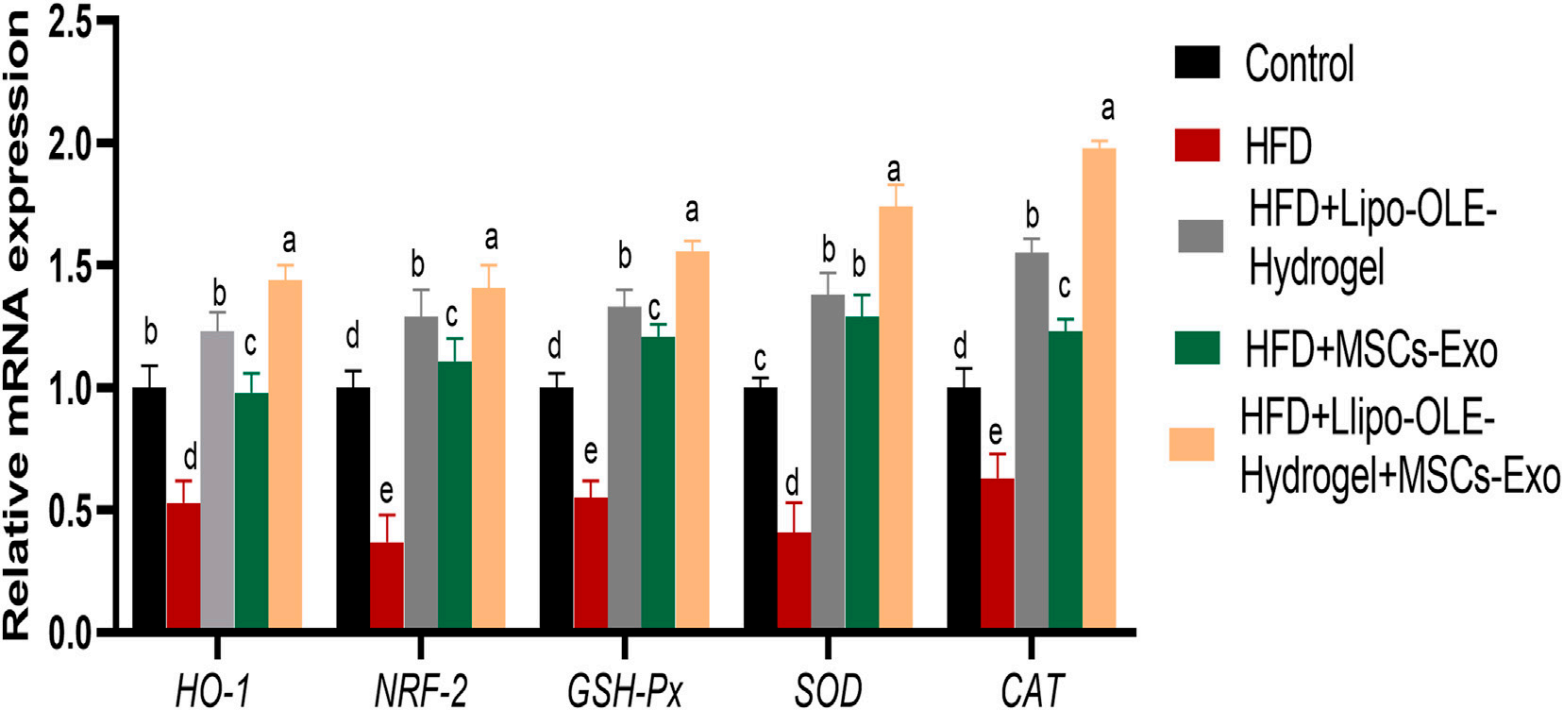
Changes in expression of antioxidant-related genes [heme oxygenase-1 (HO-1), nuclear factor erythroid 2-related factor 2 (Nrf2), glutathione peroxidase (GSH-Px), superoxide dismutase (SOD), and catalase (CAT)] in response to Lipo-OLE-Hydrogel and/or MSC-Exo treatment. Control (group fed the standard diet); HFD [group fed a high-fat diet (60% Kcal)]; HFD +Lipo-OLE-Hydrogel [group fed a high-fat diet (60% Kcal) and treated with olive-leaf-encapsulated liposomal hydrogel]; HFD + MSC-Exo (group fed a high-fat diet and treated with mesenchymal stem cell exosomes); HFD + LIPO-OLE-Hydrogel + MSC-Exo (group fed a high-fat diet and treated with olive-leaf-encapsulated liposomal hydrogel and mesenchymal stem cell exosomes). Data are expressed as means ± SE. Bars with altered letters imply significant variations (*P* < 0.05).

As illustrated in Figure 5, rats in the untreated HFD group exhibited significantly reduced expression levels of antioxidant-related genes, including HO-1, NRF-2, GPX, SOD, and CAT. In contrast, treatment with Lipo-OLE-Hydrogel + MSC-Exo in HFD-fed rats resulted in a marked upregulation of these genes, with fold increases of 1.44, 1.41, 1.56, 1.74, and 1.98, respectively.

### 3.7 Assessment of apoptotic events of HFD-fed rats in response to Lipo-OLE-Hydrogel +MSC-Exo treatment

**FIGURE 6:**
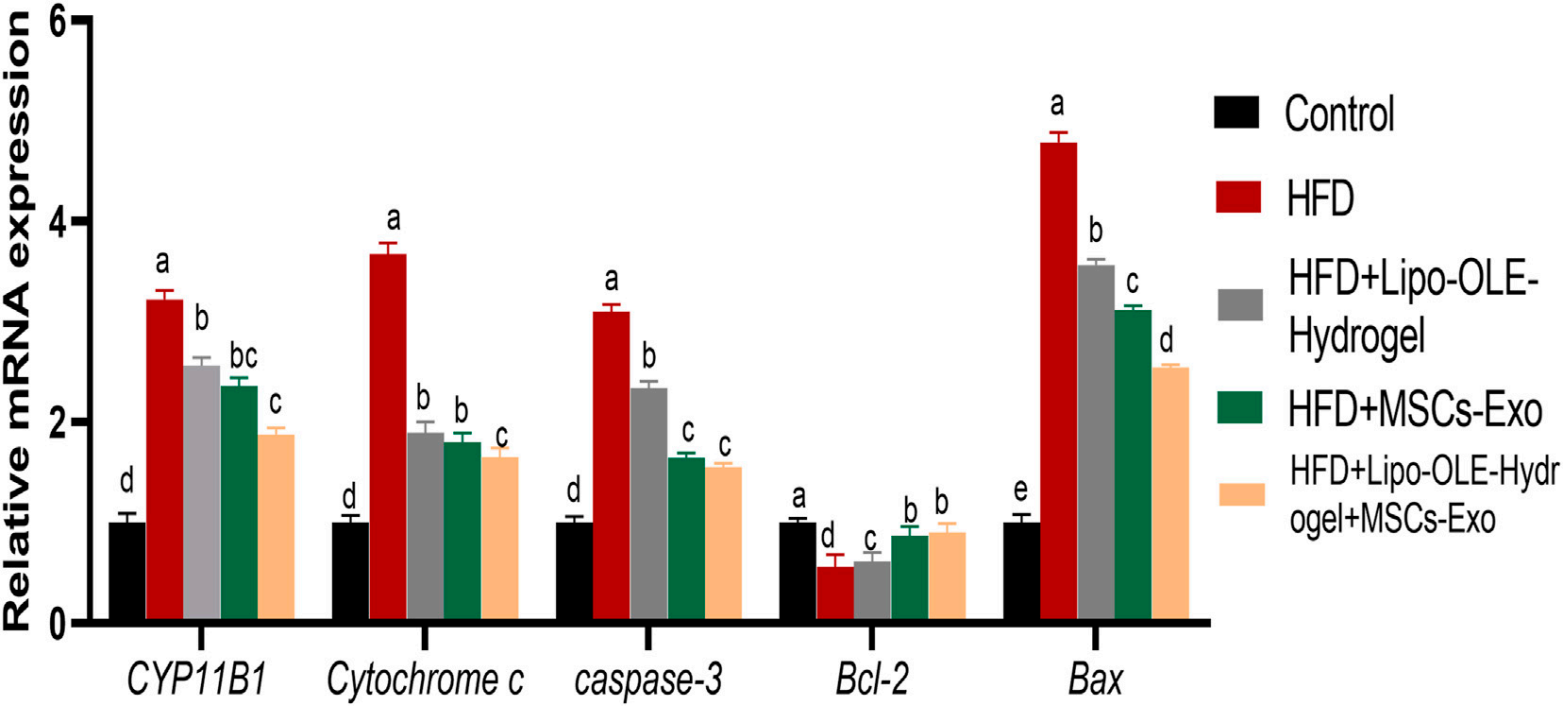
Changes cytochrome P450 family 11 subfamily B member (CYP11B1), pro-apoptotic genes [Cytochrome c, caspase-3], and anti-apoptotic genes [cell lymphoma-2 (Bcl-2) and Bcl-2 associated x protein (Bax)] in response to Lipo-OLE-Hydrogel and/or MSC-Exo treatment. Control (group fed the standard diet); HFD [group fed a high-fat diet (60% Kcal)]; HFD + LIPO-OLE-Hydrogel [group fed a high-fat diet (60% Kcal) and treated with olive-leaf-encapsulated liposomal hydrogel]; HFD + MSC-Exo (group fed a high-fat diet and treated with mesenchymal stem cell exosomes); HFD + LIPO-OLE-Hydrogel + MSC-Exo (group fed a high-fat diet and treated with olive-leaf-encapsulated liposomal hydrogel and mesenchymal stem cell exosomes). Data are expressed as means ± SE. Bars with altered letters imply significant variations (*P* < 0.05).

Figure 6 shows the expression values of *CYP11B1*, cytochrome C, caspase-3*, Bcl-2,* and *BAX.* As indicated, HFD groups that received Lipo-OLE-Hydrogel + MSC-Exo or MSC-Exo exhibited downregulated levels of caspase*-3* and *Bcl-2* when compared with untreated rats fed an HFD. Higher expression levels of BAX and cytochrome *C* in the HFD group were most prominently reduced (*P* < 0.05) in the group that received combined therapy of Lipo-OLE-Hydrogel + MSC-Exo. All HFD groups that received treatment showed clearly reduced levels of CYP11B1 compared with the HFD group without any intervention. *Bcl-2* gene expression levels were significantly upregulated (*P* < 0.05) in response to Lipo-OLE-Hydrogel + MSC-Exo or MSC-Exo compared to other therapeutic interventions.

### 3.8 Assessment of inflammation-related biomarkers and inflammasome-3 of HFD-fed rats in response to Lipo-OLE-Hydrogel+MSC-Exo treatment

**FIGURE 7:**
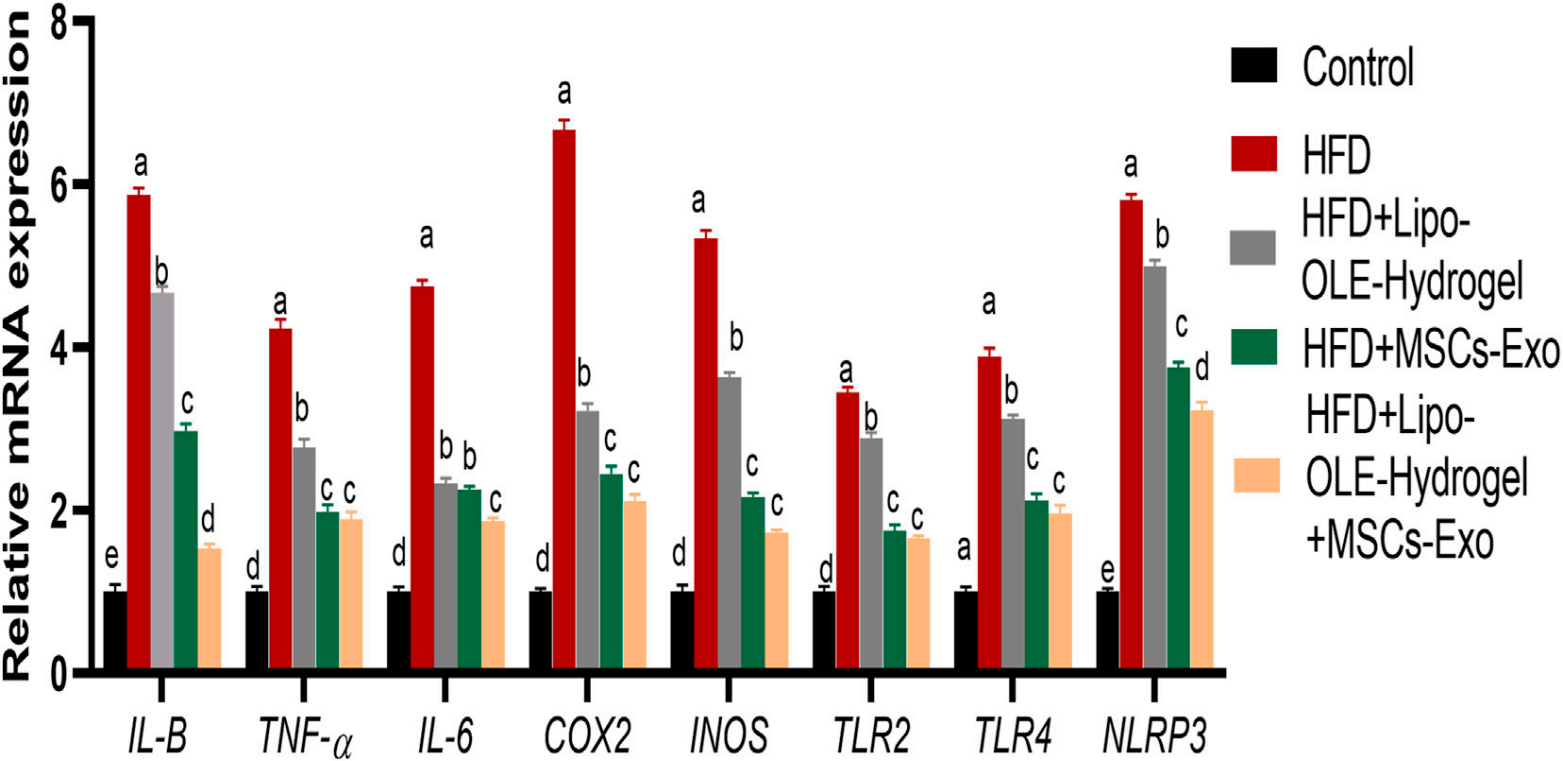
Changes in inflammatory mediators [tumor necrosis factor alpha (TNF-α), interleukin (IL-6 and IL-β)], cyclooxygenase-2 (COX-2), inducible nitric oxide synthase (iNOS), toll-like receptors (TLR2 and TLR9), pyrin domain-containing protein 3 (NLRP3) inflammasome in response to Lipo-OLE-Hydrogel and/or MSC-Exo treatment. Control (group fed the standard diet); HFD [group fed a high-fat diet (60% Kcal)]; HFD + Lipo-OLE-Hydrogel [group fed a high-fat diet (60% Kcal) and treated with olive-leaf-encapsulated liposomal hydrogel]; HFD + MSC-Exo (group fed a high-fat diet and treated with mesenchymal stem cell exosomes); HFD + Lipo-OLE-Hydrogel + MSC-Exo (group fed a high-fat diet and treated with olive-leaf-encapsulated liposomal hydrogel and mesenchymal stem cell exosomes). Data are expressed as means ± SE. Bars with altered letters imply significant variations (*P* < 0.05).

All treated groups exhibited significantly lowered expression levels of TNF-*α, IL-6*, and IL-1*β* and NLRP3, and the most prominent inhibition of these inflammatory cytokines was detected in the LIPO-OLE-Hydrogel + MSC-Exo treated group Figure 7. The expressions of *COX2*, *INOS*, *TLR2*, and *TLR9* genes were significantly increased in brain tissues in response to the consumption of the HFD. In contrast, the expression of these genes was greatly reduced (P < 0.05) in groups treated with MSC-Exo alone or in combination with Lipo-OLE-Hydrogel.

### 3.9 Assessment of endoplasmic stressor mediators of HFD-fed rats in response to Lipo-OLE-Hydrogel+MSC-Exo treatment

**FIGURE 8:**
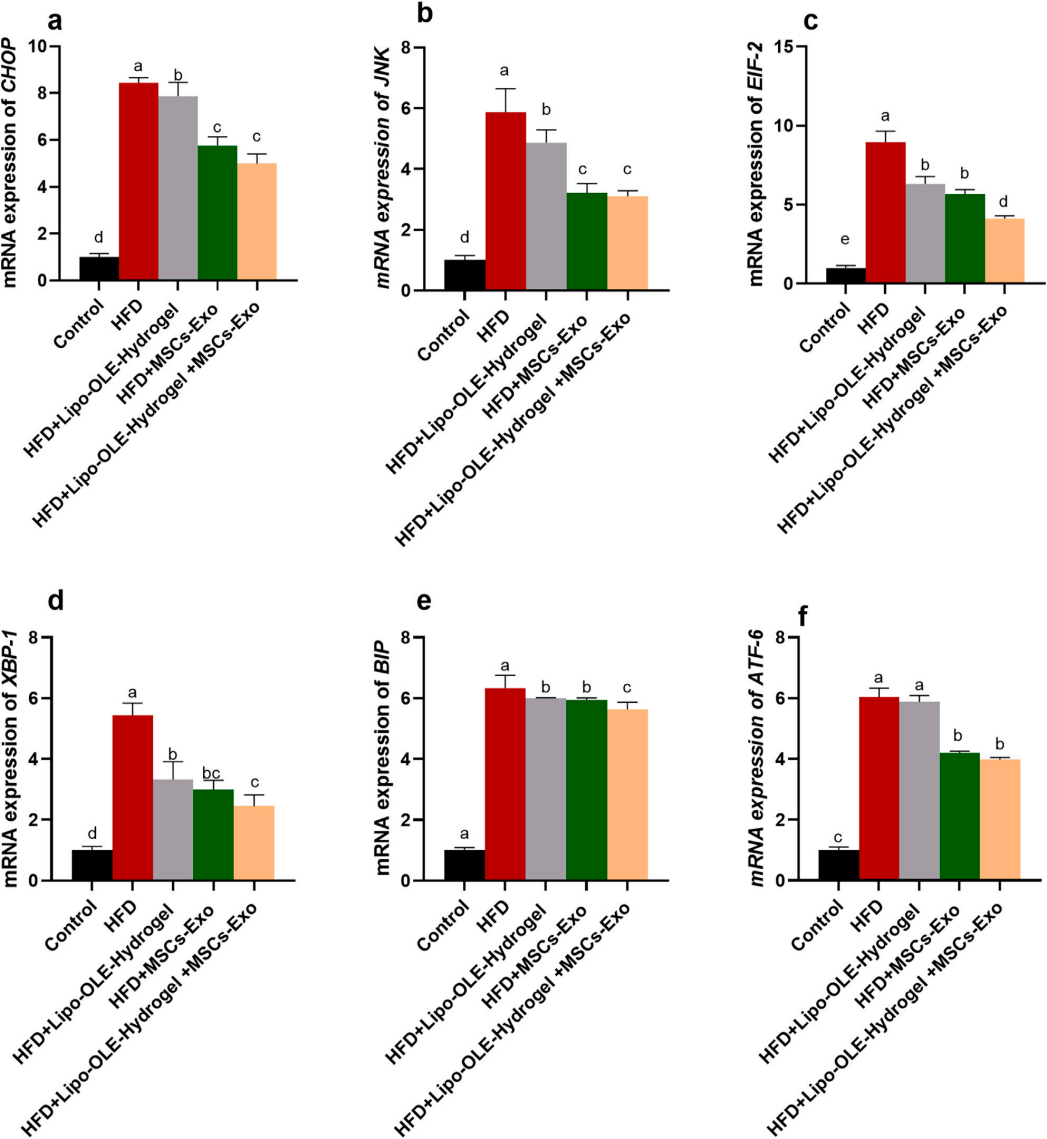
Assessment of endoplasmic stressor-related genes {C/EBP homologous protein [CHOP, **(a)**], c-Jun N-terminal kinase [*JNK*, **(b)**], eukaryotic initiation factor 2α [*eIF2α*, **(c)**], X-box-binding protein-1 [*XBP1*, **(d)**], Immunoglobulin-binding protein [*BIP*, **(e)**], activating transcription factor [ATF-6, **(f)**]} of HFD-fed rats in response to MSC-Exo + Lipo-OLE-Hydrogel treatment. Control (group fed the standard diet); HFD [group fed a high-fat diet (60% Kcal)]; HFD +LIPO-OLE-Hydrogel [group fed a high-fat diet (60% Kcal) and treated with olive-leaf-encapsulated liposomal hydrogel]; HFD + MSC-Exo (group fed a high-fat diet and treated with mesenchymal stem cell exosomes); HFD + Lipo-OLE-Hydrogel + MSC-Exo (group fed a high-fat diet and treated with olive-leaf-encapsulated liposomal hydrogel and mesenchymal stem cell exosomes). Data are expressed as means ± SE. Bars with altered letters imply significant variations (*P* < 0.05).

The expression of endoplasmic stressor-related genes (*CHOP, JNK, EIF-2, ATF-6, XBP-1*, and *BIP*) in brain tissues in response to treatment is shown in Figure 8. The highest response (*P* < 0.05) was detected in the HFD non-treated group, as verified by the remarkable upregulation in their expression levels. Notably, groups treated with Lipo-OLE-Hydrogel + MSC-Exo or MSC-Exo expressed much lower levels of CHOP, JNK, and ATF-6 when compared with the group fed an HFD. Moreover, the expression of EIF-2 and BIP reached their minimum values in the HFD group treated with the combination therapy of MSC-Exo and Lipo-OLE-Hydrogel compared to other treated groups (decreased by 4.12- and 5.64-fold vs. 8.95 and 6.34-fold in the HFD non-treated group). The expression patterns of XBP-1 were significantly downregulated in response to treatment, especially in the Lipo-OLE-Hydrogel + MSC-Exo or MSC-Exo groups (reduced by 2.99- and 2.45-fold vs. 5.43-fold in the HFD non-treated group).

### 3.10 Histopathological findings

**FIGURE 9:**
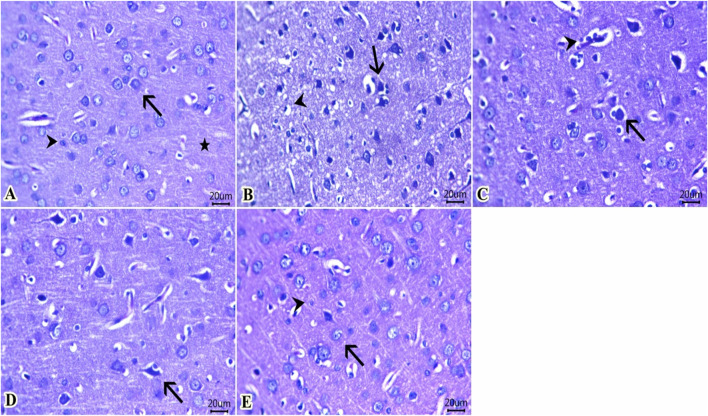
Representative photomicrograph of H&E-stained sections from cerebral cortex (Scale bar 20 μm) showing: **(A)** Normal histological structures of neuronal bodies (arrow), glia cells (arrowhead), and neuropil (star) in the control group. **(B)** A large number of pyknotic neurons surrounded by glial cells (arrow) and vacuolated neuropil (arrowhead) in rats fed an HFD without treatment. **(C)** A moderate number of shrunken neuron cell bodies (arrow) beside a minute number of lymphocytes within the Virchow–Robin space (arrowhead) were found in rats fed an HFD and treated with Lipo-OLE-Hydrogel. **(D)** A few pyknotic neurons with clear halos (arrow) were found in rats receiving an HFD and treated with MSC-Exo. **(E)** Preserved histological configurations of neurons (arrow) and glial cells (arrowhead) were found in rats fed an HFD and treated with a combined therapy of LIPO-OLE-Hydrogel and MSC-Exo.

As illustrated in Figure 9, the cerebral cortex from the control group fed the standard diet (Figure 9A) revealed normal histological structures of neuronal bodies, glial cells, neuropils, and cerebral vasculatures. The group of rats fed an HFD (Figure 9B) showed a large number of pyknotic neurons with deeply basophilic nuclei that were encircled by clear spaces and mostly surrounded by glial cells. Vacuolated neuropils that indicate demyelinated dendrites were also noticed. Meanwhile, a moderate number of shrunken neuron cell bodies beside a minute number of lymphocytes within the Virchow–Robin space were seen in the group fed an HFD and received Lipo-OLE-Hydrogel (Figure 9C). Few pyknotic neurons with clear halos were seen in the group injected with MSC-Exo (Figure 9D). Notable preserved histological configurations of the cerebral cortex were observed in a group that received a combined therapy of Lipo-OLE-Hydrogel and MSC-Exo (Figure 9E).

### 3.11 Immunohistochemical analysis

**FIGURE 10:**
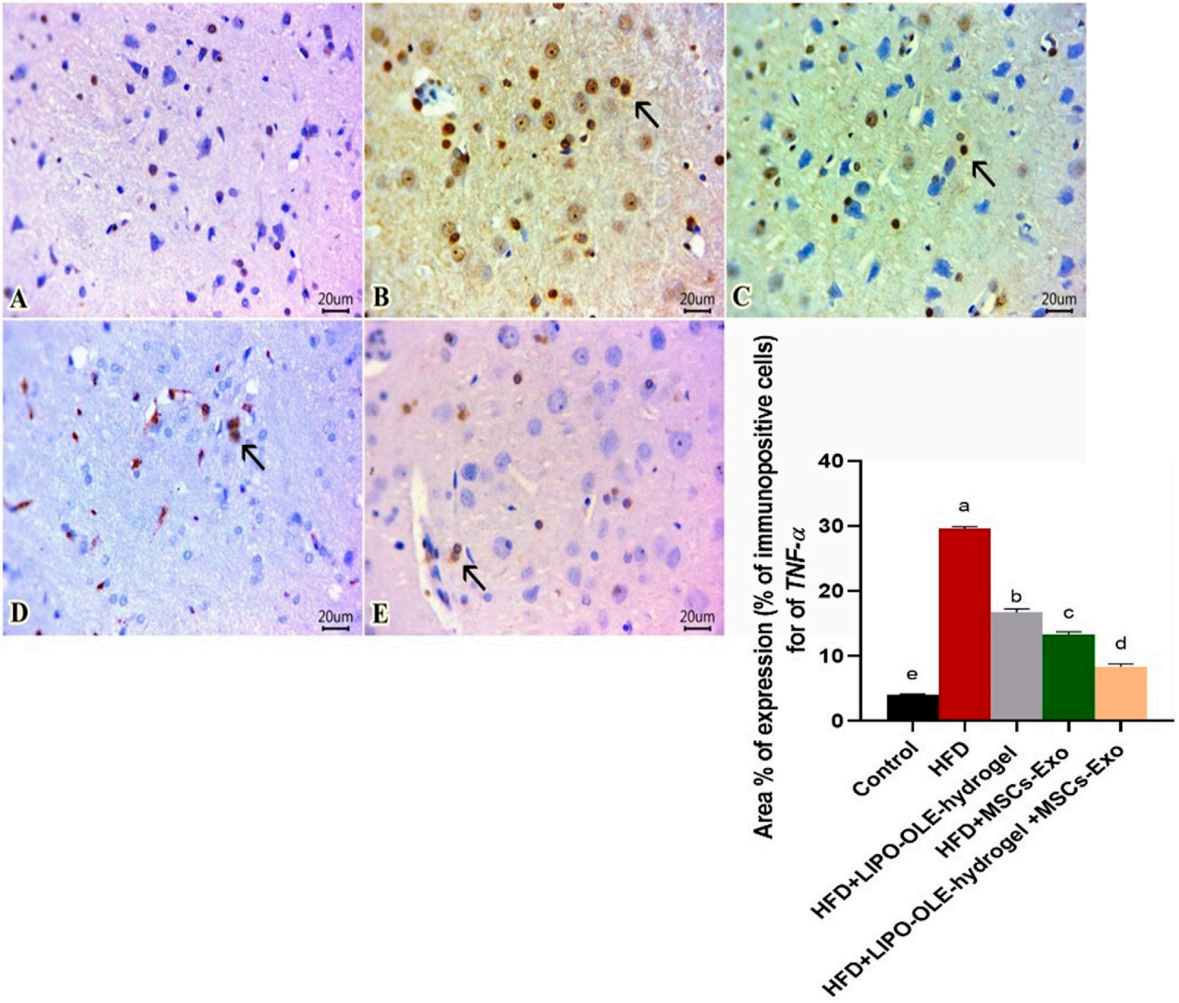
Photomicrographs of immuno-stained sections from brain tissues (scale bar 20μm) for TNF-α showing apparently negative immuno-labeled cells found in the control group fed the standard diet **(A)**. Many positive cells were noted in the group fed an HFD without treatment **(B)**. A moderate number of stained cells were observed in rats fed an HFD and treated with Lipo-OLE-Hydrogel **(C)**. Mild expression was detected in rats that received an HFD and were treated with MSC-Exo **(D)**. Few expressed cells were detected in rats fed an HFD and treated with a combined therapy of Lipo-OLE-Hydrogel and MSC-Exo **(E)**. The positively stained cells exhibited a brown color. The arrow demonstrates the positive cells. IHC counterstaining was done with Mayer’s hematoxylin.

**FIGURE 11:**
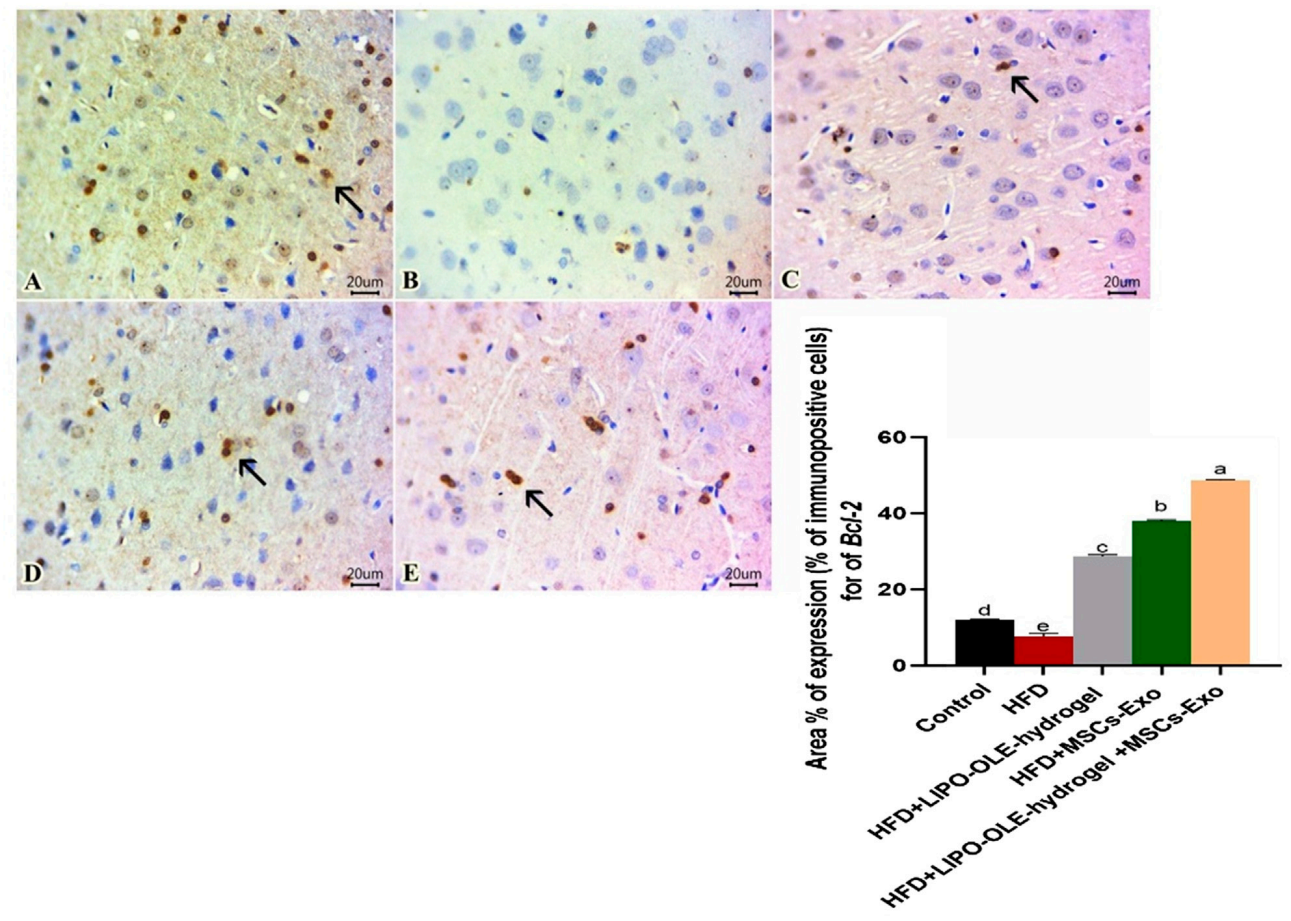
Photomicrographs of immuno-stained sections from brain tissues (scale bar 20 μm) for Bcl-2, showing numerous stained cells found in the control group fed the standard diet **(A)**. Scarcely any cells were noted in rats fed an HFD without treatment **(B)**. Few expressed cells were observed in rats fed an HFD and treated with LIPO-OLE-Hydrogel **(C)**. A small number of positive cells were detected in rats fed an HFD and treated with MSC-Exo **(D)**. A moderate number of immuno-labeled cells were perceived in rats fed an HFD and treated with a combined therapy of LIPO-OLE-Hydrogel and MSC-Exo **(E)**. Positively stained cells exhibited a brown color. The arrow demonstrates the positive cells. Immunohistochemical counterstaining was done via Mayer’s hematoxylin.

Sections of the brain immunohistochemically stained against TNF-α revealed apparently negative immunolabeling in the control group fed the standard diet (Figure 10A). However, a group of rats fed an HFD without treatment showed a large number of positively expressed cells (Figure 10B). The number of stained cells declined in all treated groups, and a moderate number of stained cells were found in the group fed HFD that received Lipo-OLE-Hydrogel (Figure 10C). Mild expression against TNF-α was detected in the group injected with MSC-Exo (Figure 10D), and few expressed cells were noted in the group that received a combined therapy of both Lipo-OLE-Hydrogel and MSC-Exo (Figure 11E). Regarding Bcl-2 expression, stained sections of the control group fed the standard diet (Figure 11A) showed many positively stained cells. However, nearly non-expressed cells were noticed in a group of HFD-fed rats that received no treatment (Figure 11B). On the other hand, there was a gradual re-establishment of Bcl2 protein labeling in all treated groups. Notably, few express cells were detected in groups fed HFD that received Lipo-OLE-Hydrogel (Figure 11C). Low numbers of positive cells were seen in the group injected with MSC-Exo (Figure 11D). A moderate number of immuno-labeled cells were found in the group that received the combined therapy of Lipo-OLE-Hydrogel and MSC-Exo (Figure 11E).

### 3.12 Statistical analysis

Statistical analyses were performed using the SPSS^®^ Statistics software (version 22, IBM Corp., USA). Normality and homogeneity of variances were assessed using the Shapiro–Wilk and Levene’s tests, respectively. Data were analyzed by one-way analysis of variance (ANOVA), followed by Tukey’s *post hoc* test for multiple comparisons. Statistical significance was considered at *P* < 0.05. All graphs were generated using GraphPad Prism (version 8, GraphPad Software Inc.).

## 4 Discussion

Obesity caused by prolonged consumption of HFD causes many life-threatening diseases, including insulin resistance, oxidative and endoplasmic reticulum (ER) stress, mitochondrial dysfunction, lipotoxicity, inflammation, hypertension, and cardiovascular mortality (Son et al., 2023). An HFD contributes to the increasing prevalence of obesity, which is associated with a heightened risk of impaired brain function, as well as behavioral and memory disturbances (de Paula et al., 2021). The impaired ability of HDF-fed animals to use fat as a metabolic fuel suggested that a large part of the higher energy intake is stored as fat, supporting findings from previous studies (Mollica et al., 2017; Cavaliere et al., 2018).

The brain is a highly stress-sensitive organ, particularly vulnerable to key pro-inflammatory constituents from dietary sources, including saturated fatty acids, cholesterol, added sugars, and refined grains (Christ et al., 2019). Changing nutritional lifestyle, aiming at weight loss and decreased fat accumulation, cannot provide sufficient treatment, especially when neurodegeneration occurs. Mesenchymal stem cells derived from exosome-based therapy (MSC-Exo) not only mediate communication among cells but can also be engineered to deliver specific substances. MSC-derived exosomes can mediate numerous cellular events, such as cell death, immune response, glucolipid metabolism, and oxidative stress, contributing to tissue repair (Yu et al., 2014; Li et al., 2021). Because exosomes can cross the blood–brain barrier (Kalluri and LeBleu, 2020), their implication in the treatment of neurodegenerative diseases has received recent attention (Soria et al., 2017). Adverse health consequences beyond HFD consumption include a higher incidence of brain oxidative stress and impairment of brain mitochondrial functions. However, the underlying mechanisms by which MSC-derived exosomes (MSC-Exo) exert their effects are not yet fully understood.

Olive leaf extract, with an abundant content of phenolic antioxidants, plays an important role in the treatment of obesity-related metabolic disorders via activating fatty acid oxidation and inhibiting lipogenesis (Mikami et al., 2021). However, due to its low solubility and sensitivity to environmental conditions, it needs an effective vehicle to enhance its usability and bioavailability (González-Ortega et al., 2021). In this sense, the development of effective novel evidence-based therapy, combining nanotechnology and phytotherapy, for neuroinflammation, and in particular, those with long-lasting effects, is urgently required. The generation of reactive oxygen species (ROS) is one of the most prevailing mechanisms for impeding MSCs’ stemness and triggering cellular senescence. Addressing oxidative stress or triggering antioxidant mechanisms via using phytotherapy of antioxidant power is one of the chosen approaches for improving the therapeutic efficacy of MSCs in further clinical applications. Achieving a therapeutic approach comprising the administration of LIPO-OLE-Hydrogel together with injection of MSC-Exo as a novel combined therapy (LIPO-OLE-Hydrogel + MSC-Exo) successfully reversed the progression of HFD-induced neuroinflammation.

The current study showed that an HFD increased body weight gain and body lipid levels, which is in accordance with Cavaliere et al. (2018) and Labban et al. (2020). Administration of LIPO-OLE-Hydrogel resulted in decreased body weight and fat mass in rats fed an HFD, which is in agreement with Djeziri et al. (2018). Oleanolic acid, a bioactive metabolite of olive leaf extract, causes a rise in β-oxidation and energy generation, resulting in fat reduction and anti-obesity effects (Watanabe et al., 2006). The response to HFD progression includes hyperglycemia and impairments in glucose clearance, which align with Dutheil et al. (2016). Dietary intervention with Lipo-OLE-Hydrogel may be an effective alternative for weight loss and weight management, decreasing the risk and progression of chronic diseases.

*In vitro* and animal studies suggest that the potential mechanisms of LIPO-OLE-Hydrogel for additional fat loss involve the phenols in LIPO-OLE-Hydrogel reducing adipocyte proliferation and increasing thermogenesis through the activation of brown adipose tissue (Melguizo Rodríguez et al., 2021). The delayed weight gain and reduced glucose tolerance supported the protective role of Lipo-OLE-Hydrogel + MSC-Exo against the metabolic syndrome associated with HDF-induced obesity. Additionally, the lowered level of glucose during the experimental period post-treatments with MSC-Exo could be attributed to the regulatory role of MSC-Exo on insulin sensitivity via modulating inflammation or by direct interaction with insulin-responsive organ mechanisms (Kumar et al., 2021). MSC-Exo facilitates the oxidation of fatty acids and inhibits the synthesis of fatty acids through lowering lipid metabolism-correlated proteins expression and thus inhibits lipid deposition in non-alcoholic fatty liver diseased rats (Cheng et al., 2021). Exosomes exert remarkable effects on lipid metabolism, including the synthesis, transport, and degradation of lipids (Wang et al., 2020). The delayed weight gain observed in rats fed a high-fat diet (HFD) and treated with the combined therapy of Lipo-OLE-Hydrogel and MSC-Exo could be related to higher energy expenditure associated with MSC-Exo injection (Castaño et al., 2022). The novel therapeutic approach involving the administration of Lipo-OLE-Hydrogel demonstrates protective effects on the progression of insulin resistance and reduces daily weight gain, likely due to its high polyphenolic content.

Supplementation with olive leaf extract improved insulin sensitivity by 15% (Haidari et al., 2021). Lipo-OLE-Hydrogel enhances insulin-stimulated glucose transport in adipocytes, suggesting that it may improve insulin sensitivity (Ryan et al., 2000). It has also been proposed that the anti-glycemic effects of OLE result from its ability to prevent starch digestion and glucose uptake, stimulate hepatic glycogen synthesis, and inhibit pancreatic α-amylase activity (Alshaer et al., 2025). Our interesting findings regarding improved glucose tolerance and insulin sensitivity after exosome therapy could be attributed to their role in attenuating HFD-induced obesity, primarily through their protective effect on islets by reducing macrophage infiltration and inflammation (Chen W. et al., 2016). The reduction in hyperglycemia observed in HFD-fed rats treated with exosomes could be due to increased insulin sensitivity, potentially through the modulation of AKT phosphorylation levels and enhanced glucose uptake in AML12 cells (Lei et al., 2021). MSC-Exo treatment mitigates the glucose and lipid metabolism dysfunctions via depressing fat mass and obesity-associated gene expression, contributing to non-alcoholic fatty liver disease management (Tamimi et al., 2024). Accordingly, our data indicated an increase in serum leptin levels in HFD-fed rats that confirmed obesity in these groups.

Leptin, a hormone involved in regulating food intake and body weight homeostasis, is typically elevated in obesity, leading to a condition known as leptin resistance (Labban et al., 2020). Hyperleptinemia has been shown to influence pointedly the obesity development and its correlated neurological disorders (Hristov et al., 2019). Obese rats were reported to typically develop leptin resistance (Scarpace et al., 2000). This finding can also be corroborated by the most recent research of Mzhelskaya et al. (2020) and Palacios-Rodriguez et al. (2024). In the context of HFD-induced obesity, leptin’s anorexigenic impact on hypothalamic neurons is diminished, resulting in an increase in appetite and increased fat mass. A diet enriched in polyphenol-based olive byproducts reduced serum levels of leptin (Carpi et al., 2019). Herein, reduced levels of leptin following a combined therapy of Lipo-OLE-Hydrogel + MSC-Exo indicated their modulatory role on serum leptin levels. OLE can be suggested to improve lipid profile, glycemic status, and free fatty acids levels in serum by body weight reduction, increasing serum adiponectin, and decreasing serum levels of leptin (Haidari et al., 2021).

In the current study, HFD-induced hyperlipidemia was detected by a significant increase in LDL, triglyceride, and cholesterol levels. Application of Lipo-OLE-Hydrogel and MSC-Exo not only helped to maintain a healthy body weight but also improved the blood lipid profile. The observed effects of MSC-Exo on reducing hypercholesterolemia and increased triglyceride levels in rats with HFD-induced obesity could be attributed to their reparative effect on hepatic tissue and the enhancement of liver function in promoting lipid oxidation, which aligns with previous findings by Rashidi et al. (2023). Our data were in line with Mikłosz et al. (2022), who stated that MSC-Exo-based therapy was sufficient to reduce body fat mass in diet-induced obesity animals and suppress the increase in body weight, which could be related to the promotion of insulin production, improvement of insulin resistance, and regulation of hepatic metabolism. In the same line, consumption of Lipo-OLE-Hydrogel has been shown to lower weight gain over time (Razquin et al., 2009; Shen et al., 2014) and LDL-C (Solá et al., 2011), increase HDL-P (Flynn et al., 2017), and improve insulin sensitivity, which is attributed to its higher phenolic content. These boosted outcomes related to Lipo-OLE-Hydrogel in the current study are mainly attributed to its encapsulation in nano materials that could enhance its stability and functionality (González-Ortega et al., 2021).

In our study, animals fed a high-fat diet (HFD) and treated with MSC-Exo or the combined Lipo-OLE-Hydrogel + MSC-Exo therapy exhibited improved glucose clearance and reduced hyperlipidemia compared to those treated with Lipo-OLE-Hydrogel alone. These findings suggest that Lipo-OLE-Hydrogel may not be sufficient on its own to fully mitigate obesity-related metabolic disorders, but its effectiveness is significantly enhanced when combined with MSC-Exo therapy.

Growing evidence demonstrates that the time-course effects of HFD-induced obesity significantly impact learning, memory processes, and mood-related behaviors (Pratchayasakul et al., 2011). In this study, a shift to a high-fat diet (HFD) significantly impacted brain function, mood-related behaviors, and performance in memory-related tasks, as evident in altered open-field test outcomes. Notably, impaired glucose regulation, reflected by increased blood glucose levels—particularly after 14 weeks of HFD intake—was associated with deficits in learning, memory, and behavior. We observed pronounced behavioral changes linked to anxiety, consistent with prior studies in rats and mice, which have shown that high-energy diets can lead to anxiety-like behaviors through increased activity of the hypothalamic–pituitary–adrenal (HPA) axis (Pratchayasakul et al., 2011). Notably, the behavioral deficits induced by HFD were rapidly reversed by the combined therapy of Lipo-OLE-Hydrogel and MSC-Exo, as evidenced by improvements in the novelty-suppressed feeding test (NSFT) and home cage feeding (HCF) assessments. This can be explained by the effective properties, stability, and release of OLE active principles from OLE in liposomal hydrogel (Istenič et al., 2016).

It is well understood that dopamine and serotonin play an essential role in homeostatic signaling as neurotransmitters (Tuominen et al., 2015). Based on previous studies in humans and rodents, modulation (either inhibition or stimulation) of these neurotransmitters has been linked to changes in feeding behavior, appetite regulation, energy expenditure, and reward-based learning, as demonstrated in studies such as Halford et al. (2005) and Palmiter (2008). In the case of food-related processes, increased 5-HT is generally thought to suppress feeding behavior and serve as a satiety signal in mammals (Yabut et al., 2019). The notable increase in amounts of brain dopamine in obese rats noted in this study can be clarified by the fundamental contribution of the dopamine system in controlling hyperphagia, response to high-energy diets, and the pathophysiology of obesity (Geiger et al., 2009). We hypothesized that the alterations in the feeding behavior after eating an HFD associated with obesity are a consequence of variations in the pivotal serotonin and dopamine systems, which align with Halford, Harrold, Lawton, and Blundell (2005). The unusual decrease in brain serotonin (5-HT) in HFD-triggered obese rats, as reported here, is reinforced by recent work of Haleem and Mahmood (2021), in which a significant reduction in serotonin was observed in obese subjects. Their findings further support the neurochemical theories underlying disrupted feeding behavior in obesity, highlighting the role of neurotransmitters such as serotonin in the regulation of appetite and energy balance. Bautista et al. (2019) illustrated the association between glutamate and obesity-linked markers of inflammation, dyslipidemia, and oxidative stress. The significant increase in brain glutamate reported in the present study was supported by the work of Valladolid-Acebes et al. (2012). This supports the hypothesis that elevated levels of nutritional glutamate play an etiological role in the development of obesity. This concept underscores the importance of developing weight management medications that target brain neurotransmitters responsible for regulating food intake.

These research findings provide new insights into how high-fat consumption influences neural mechanisms and their potential role in exacerbating non-homeostatic eating behaviors. The synergistic effect of combined Lipo-OLE-Hydrogel + MSC-Exo on the amelioration of brain neurotransmitter impairment suggests their effective role in the treatment of neurocognitive disorders, aligning with a previous study (Harrell et al., 2021). In addition, inflammation is a central feature of obesity and altered glucose tolerance, impacting not only the blood and peripheral systems but also the central nervous system. Pro-inflammatory cytokines interact with multiple signaling pathways, playing a key role in mood regulation and contributing to the development of depression (Haroon et al., 2012). It has also been found that high-fat diet consumption (HFD) can evoke an inflammatory response (Cavaliere et al., 2018). Additionally, significant inflammatory damage following ingestion of HFD was detected in brain tissues of the non-treated group, as displayed by scattered degenerations, a decrease in the viable pyramidal cells in areas of the hippocampus, a decrease in the thickness of granular cells in the dentate gyrus, and observed signs of apoptosis (Hazzaa et al., 2020; Mengr et al., 2023). Accordingly, cognitive impairment, such as impaired glucoregulation (Mielke et al., 2006), increased brain inflammation, and alteration in blood–brain barrier permeability (Cordner and Tamashiro, 2015), has been attributed to consumption of an HFD (Xia et al., 2015).

We observed increased expression levels of inflammatory cytokines, which are associated with higher levels of oxidative stress biomarkers in the brain tissues of HFD rats. This confirms that the neuroinflammation observed in the diet-induced obesity animal model may be linked to the increased expression of inflammatory markers. This could be attributed to the reduced ability of HFD-fed animals to utilize fat as a metabolic fuel, leading to the accumulation of toxic lipid species that trigger ROS overproduction and inflammation (Mollica et al., 2017). NF-κB is a transcription factor implicated in the regulation of a wide range of genes related to apoptosis, inflammation, and immune response, and the expression of many chemokines, cytokines, and other inflammatory mediators, including *Nos2*, *Cox2*, and *TNF-α*, is under the control of *NF-κB* activation (Duval et al., 2015). Toll-like receptors (TLRs) are danger-associated molecular pattern receptors that also interact with insulin, inflammation, and mTORC1 signaling pathways (Ghasemi et al., 2013). These receptors serve a variety of functions involving the inflammasome, protein synthesis regulation, inflammatory cytokine expression, apoptosis, and cell survival (Hanke and Kielian, 2011). Data from another animal study further demonstrated that HFD triggers TLR4 and endoplasmic reticulum stress signaling pathways (Zhang et al., 2008; Wang X. et al., 2012).

In the current study, inflammatory responses in brain tissues were increased in obese rats, including the upregulation of TLR4 and NF-κB, as well as the production of pro-inflammatory cytokines (IL-6, IL-1β, and TNF-α). This finding is also linked to the significant alterations in serotonin, dopamine, and glutamate, which were discussed earlier in the study as markers of neuronal damage, and is consistent with previous research of Labban et al. (2020). In the current study, progressive inflammatory responses subsided more prominently in the group that received Lipo-OLE-Hydrogel + MSC-Exo. This was attributed to the health-promoting and anti-inflammatory effects of Lipo-OLE-Hydrogel, resulting from its increased absorption rate, higher bioavailability, and increased permeation through the intestine after encapsulation in an appropriate nano-delivery system (Munin and Edwards-Lévy, 2011). Additionally, mesenchymal stem cell-derived extracellular vesicle (MSC-EV) treatment suppressed neuroinflammation by inhibiting NF-κB activation and expression (Xin et al., 2021).

Oxidative stress, resulting from an imbalance between reactive oxygen species (ROS) and the endogenous antioxidant defense system, plays a pivotal role in neurodegeneration. This is particularly significant in the brain, which is characterized by high oxygen consumption, increased ROS production, a high content of polyunsaturated fatty acids, and relatively low antioxidant defenses (Angeloni et al., 2019). Throughout the high-fat feeding, the mismatch between uptake and utilization of fatty acids can lead to toxic lipid accumulation, triggering ROS overproduction and inflammation. Additionally, oxidative stress contributes to brain damage and induces cell injury with impaired learning and memory (Hazzaa et al., 2020). Importantly, this central inflammation in brain tissues can actually contribute to leptin and insulin resistance, favoring weight gain and maintaining an increased body weight, which agrees with Posey et al. (2009). The current study indicated that Lipo-OLE-Hydrogel + MSC-Exo improved brain function by suppressing the toxic effect of lipids via downregulating inflammatory markers and oxidative stress-related genes and biomarkers in the brain (as shown by a reduction in MDA, H_2_O_2_, and ROS content with significant increases in TAC and GSH-PX content in brain tissues). MDA is one of the final products in the peroxidation of polyunsaturated fatty acids by the cell, and its higher concentration in brain tissues is an indicator of oxidative stress (Labban et al., 2020).

In this context, when mesenchymal stem cells (MSCs) are engrafted into tissues with elevated levels of inflammatory cytokines, they adopt an immunosuppressive phenotype and produce a variety of immunosuppressive factors, leading to the inhibition of inflammatory immune cells (Gazdic et al., 2015). The beneficial effects of MSCs are attributed to their migration toward inflamed areas, driven by the expression of chemokine receptors. These receptors help inhibit or limit inflammatory responses and promote anti-inflammatory pathways (Wang S. et al., 2012). In addition to differentiation and integration into the damaged tissue, the mode of action of MSCs when implanted in the injured region could involve several mechanisms: immunomodulatory and anti-inflammatory properties, induction of neurogenesis, secretion of neurotrophic factors, promotion of axon growth, enhancement of synaptic and cortical connections, and reduction of apoptosis and oxidative stress (Angeloni et al., 2020). MSCs are able to reduce H_2_O_2_-induced inflammatory factor, downregulate intracellular oxidant factor (MDA), upregulate intracellular antioxidant factor (SOD), and increase neurotrophin expression (Cui et al., 2017), in addition to increasing GSH and SOD activity and decreasing MDA activity (Chang et al., 2021; Yang et al., 2013).

Cells respond to oxidative stress by inducing antioxidant enzymes that can neutralize ROS, counteracting cell damage (Otsuki and Yamamoto, 2020). In particular, nuclear factor erythroid 2-related factor 2 (Nrf2) increases the expression of these antioxidant enzymes, thanks to its binding to a specific sequence in the promoter region of these genes called antioxidant response element (*ARE*) (Nam and Keum, 2019). Nrf2 is a key transcription factor involved in protecting against ROS generation and oxidative damage (Gao et al., 2020). Once Nrf2 is translocated to the nucleus, it induces the expression of genes including HO-1, SOD, and GSH, to counteract oxidative stress and maintain redox status balance (Kopacz et al., 2022). In this study, we consistently observed that upregulation of Nrf2 significantly reduced the inflammatory response and ROS production, while increasing the expression of antioxidant enzyme-related genes in brain tissues following treatment with Lipo-OLE-Hydrogel + MSC-Exo, highlighting their antioxidant effect.

In the current study, the optimal functionality of the novel nanocomposite formulated with Lipo-OLE-Hydrogel is linked to its higher biodegradability and controlled release of active biomolecules in the targeted area, which accelerates its curing properties. The benefits of combining natural antioxidants, such as Lipo-OLE-Hydrogel, with exosome injection are to reduce oxidative stress and create a balance between oxidants and antioxidants in the brain environment. This can be achieved via multiple pathways, such as regulating the formation of free radicals, the scavenging of excess radicals, and by interfering in the free radical chain reaction cascade, thereby eliminating the oxidative damage that may contribute to better media for survival, proliferation, and therapeutic effectiveness of MSC-Exo (Zheng et al., 2023).

Recent studies have shown that MSC-derived exosomes promote functional recovery, support neurogenesis, and reduce neuroinflammation in rats after traumatic brain injury, due to their protein composition, lipids, miRNAs, and mRNAs, which play a crucial role in cell-to-cell communication (Yang et al., 2017). The prominent impact of MSC-Exo on the inflammatory response in HFD-fed rats was attributed to their role in inhibiting macrophage inflammatory response (Zhao et al., 2018), indicating their role in the treatment of obesity-associated inflammation. Additional effects of Lipo-OLE-Hydrogel are attributed to its polyphenols, such as oleuropein, hydroxytyrosol, tyrosol, and caffeic acid, which play a crucial role in providing antioxidant and anti-inflammatory effects (Covas et al., 2006). The principal mechanisms of action of polyphenol-based olive byproducts in inflammatory response are a decrease in NF-κB activation, a decrease in LDL oxidation, and an improvement in insulin resistance (Assy et al., 2009). In brain tissues, the biological actions of phenols in Lipo-OLE-Hydrogel have been attributed to their ability to scavenge reactive species or through their possible influences on intracellular redox status (Pollard et al., 2006). In addition, olive leaf phenols have been shown to have some neuroprotective effects against Alzheimer’s disease, Parkinson’s disease, and peripheral neuropathy (Monti et al., 2011). Some findings suggested that Lipo-OLE-Hydrogel had a beneficial effect on learning and memory deficits. It was also observed that an HFD activated apoptosis in brain tissues, as indicated by the upregulation of pro-apoptotic genes caspase-3 and cytochrome c, alongside the downregulation of the anti-apoptotic gene Bcl-2, which is consistent with findings of Crespo et al. (2012), Liu and Wang (2011), and Palacios-Rodriguez et al. (2024). These favorable effects of OLE were attributed to its incorporation in a nano formulation comprised of liposomes that can prolong the release of these active substances, thus optimizing its therapeutic delivery (González-Ortega et al., 2021).

Oxidative stress stimulates the expressions of several genes involved in cell death through apoptosis (Crespo et al., 2012). The Bcl-2 family, which incorporates Bcl-2 and Bax, has a significant role in managing apoptosis. Bcl-2 is considered a pro-survival signal, and Bax is a pro-apoptotic member as it binds and antagonizes Bcl-2 actions (Becker et al., 2013). Our data indicate that Lipo-OLE-Hydrogel + MSC-Exo significantly upregulated Bcl-2 expression, as supported by immunostaining, while downregulating the pro-apoptotic genes Bax, cytochrome c, and caspase-3. These findings highlight their key role in attenuating brain apoptosis. These results are consistent with previous studies suggesting that the modulation of brain apoptosis is associated with the mitigation of oxidative stress (Kruidenier et al., 2003).

Endoplasmic reticulum stress appears in brain regions during obesity and has been implicated in the development of obesity (Çakir et al., 2013). Excessive ER stress can lead to apoptosis and neurodegeneration (Tabas and Ron, 2011) and eventually brain atrophy. ER stress can also exacerbate cellular inflammatory pathways and cause ROS production, which in turn can exaggerate ER stress (Hotamisligil, 2010). Thus, inflammation-related ER stress may also contribute to neuronal dysfunction either directly or by modulating oxidative stress and inflammation. The current study proposed a tight correlation between expression of ER stress and inflammation-related genes, as evidenced by the overexpression of *eIF2α, ATF6, PERK, JNK, XBP1*, and *CHOP* mRNA levels in brain tissues in HFD-fed rats. These findings are in agreement with higher expression of caspase-3 and cytochrome C in non-treated HFD rats. Moreover, prolonged activation of ER stress in rats fed an HFD consecutively leads to higher expression of NLRP3 inflammasomes and an increase in pro-inflammatory cytokine levels (Oslowski et al., 2012). HFD-induced prolonged unfolded protein response (UPR) activation can induce apoptosis and PERK-mediated phosphorylation of eIF2α, which may contribute to cell death by inhibiting the synthesis of pro-survival proteins. One of the downstream targets of PERK, the transcription factor CHOP, can repress Bcl-2 expression (McCullough et al., 2001). The pro-apoptotic action occurs mainly through activation of the PERK-eIF2α-ATF6-CHOP-Bcl-2 pathway (Lin et al., 2007), which suppresses the synthesis of anti-apoptotic Bcl-2 proteins.^.^Bcl-2 is an anti-apoptotic protein that prevents the release of cytochrome c from mitochondria by inhibiting apoptosis-induced mitochondrial pore formation. Translocation of cytochrome c from the mitochondria to the cytoplasm is a key event in the activation of caspase-3 and the induction of apoptosis (Lin et al., 2007). The downregulation of ER-related genes was more prominent in the group treated with MSC-Exo or the combination therapy of Lipo-OLE-Hydrogel + MSC-Exo. This effect could be attributed to the role of exosomes in upregulating Bcl-2 expression and inhibiting caspase-3 activation. Under ER stress, PRP-derived exosomes inhibit CHOP-mediated suppression of Bcl-2 protein expression through the Akt/Bad/Bcl-2/Caspase-3 signaling pathway, thereby protecting cells from apoptosis (Tao et al., 2017). MSC-exosomes can exert anti-inflammatory and neuroprotective effects by inhibiting the expression of NF-kB and endoplasmic reticulum-related genes (Ren et al., 2020).

The above-mentioned findings were also supported by the synergistic healing effect of Lipo-OLE- with MSC-Exo on brain tissue regeneration in the histological picture that is evinced by regeneration and normal architecture of brain tissues in such groups. The enhanced therapeutic effect of combining Lipo-OLE-Hydrogel with MSC-Exo injection can be attributed to Lipo-OLE-Hydrogel’s ability to alleviate neuroinflammation by reducing oxidative stress. This creates a more favorable microenvironment by suppressing cellular senescence, thereby supporting the proliferation and migration of MSC-Exo and enhancing their overall therapeutic efficacy (Zheng et al., 2023).

## 5 Conclusion

The neuroprotective effect of Lipo-OLE-Hydrogel, when combined with the regenerative effect of MSC-Exo, exerted a promising curative role against HFD-induced neuroinflammation progression. Taken together, our findings indicate that the Lipo-OLE-Hydrogel + MSC-Exo therapy provides effective neuroprotection and reduces the deleterious impact of an HFD by ameliorating the accumulated oxidative and endoplasmic stress mediators in brain tissues, thereby reducing the excessive inflammatory response and associated damage. More precisely, MSC-Exo, together with Lipo-OLE-Hydrogel, resulted in considerable improvement in memory and behavioral deficits caused by an HFD. Our findings, therefore, open new avenues for potential treatments of brain dysfunction associated with the adverse effects of HFD consumption by combining natural protective and regenerative agents.

## Data Availability

The data presented in this study is available on request from the corresponding author.
