# Supplementary material for: Crosstalk between MSC-extracellular vesicles and Olea europaea leaf extract in encapsulated liposomal hydrogel: attenuation of neuroinflammation and brain neurotransmitter and memory impairment associated with obesity-induced high-fat diet
Source: Front Pharmacol. 2025 Nov 3;16:1621092. doi: 10.3389/fphar.2025.1621092 (PMC12620834; doi:10.3389/fphar.2025.1621092)
Supplement: Supplementary file 1 [file DataSheet1.pdf]

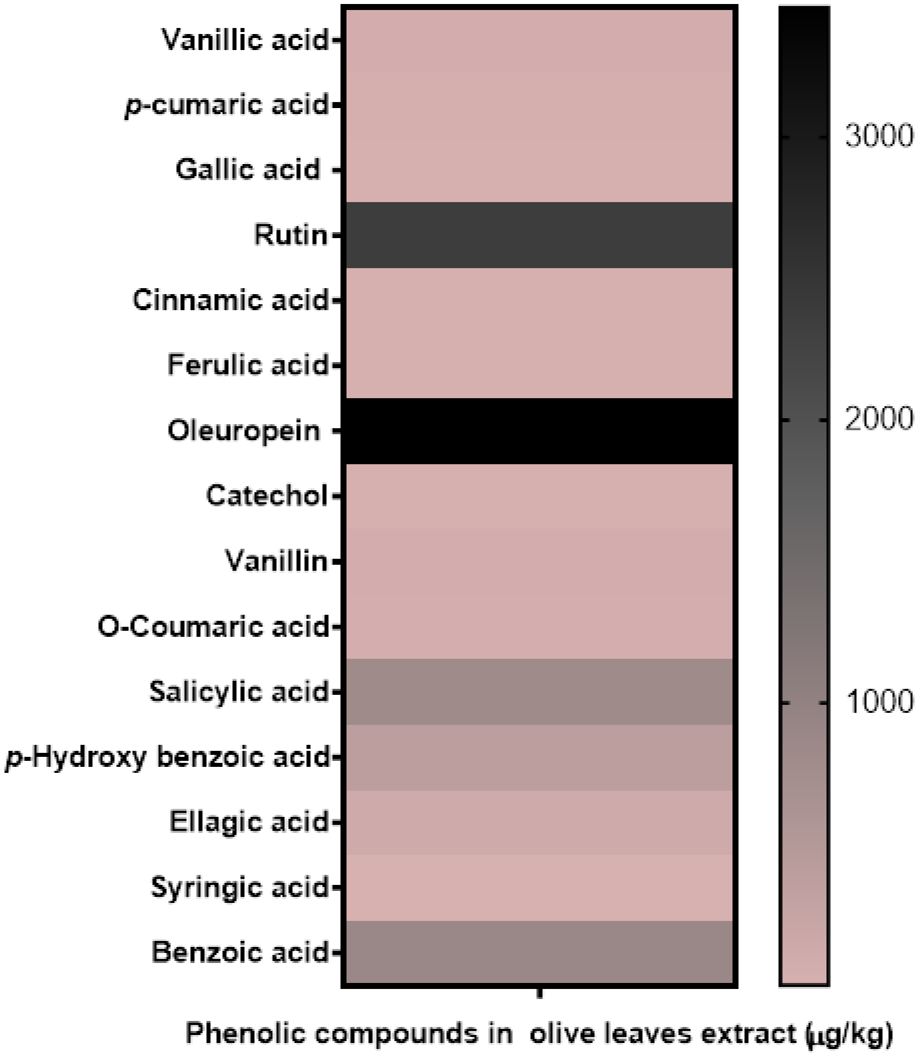

S1: Analysis of phenolic compounds in olive leaves extract (µg/kg) by high liquid gas chromatography.
